# NY-ESO-1-specific T cell receptor-engineered T cells and Tranilast, a TRPV2 antagonist bivalent treatment enhances the killing of esophageal cancer: a dual-targeted cancer therapeutic route

**DOI:** 10.1186/s12935-024-03249-w

**Published:** 2024-02-09

**Authors:** Obed Boadi Amissah, Wenfang Chen, Jean de Dieu Habimana, Yirong Sun, Lihui Lin, Yujie Liu, Ling Wang, Zhaoming Liu, Omar Mukama, Rajesh Basnet, Hohua Liu, Junyi Li, Xuanyan Ding, Lingshuang Lv, Min Chen, Yalin Liang, Rongqi Huang, Zhiyuan Li

**Affiliations:** 1 CAS Key Laboratory of Regenerative Biology, Guangdong Provincial Key Laboratory of Stem Cell and Regenerative Medicine, Guangzhou Institutes of Biomedicine and Health, Chinese Academy of Sciences, Guangzhou, 510530 China; 2 University of Chinese Academy of Sciences, 19 Yuquan Road, Shijingshan District, Beijing, 100049 China; 3 GIBH-HKU Guangdong-Hong Kong Stem Cell and Regenerative Medicine Research Centre, GIBH-CUHK Joint Research Laboratory On Stem Cell and Regenerative Medicine, Guangzhou Institutes of Biomedicine and Health, Chinese Academy of Sciences, Guangzhou, 510530 China; 4 School of Life Sciences, University of Science and Technology of China, Hefei, 230026 China; 5 Guangdong Provincial Key Laboratory of Protein Function and Regulation in Agricultural Organisms, College of Life Sciences, South China Agricultural University, Guangzhou, 510642 China; 6 GZMU-GIBH Joint School of Life Sciences, Guangzhou Medical University, Guangzhou, 511436 China; 7 Department of Anatomy and Neurobiology, Xiangya School of Medicine, Central South University, Changsha, 410013 China

**Keywords:** Adoptive therapy, Cytotoxic T lymphocytes, Esophageal cancer (EC), NY-ESO-1 antigen, T cell receptor-engineered T cell (TCR-T), Tranilast

## Abstract

**Background:**

Esophageal cancer (EC) is a global canker notorious for causing high mortality due to its relentless incidence rate, convoluted with unyielding recurrence and metastasis. However, these intricacies of EC are associated with an immoderate expression of NY-ESO-1 antigen, presenting a lifeline for adoptive T cell therapy. We hypothesized that naturally isolated higher-affinity T cell receptors (TCRs) that bind to NY-ESO-1 would allow T lymphocytes to target EC with a pronounced antitumor response efficacy. Also, targeting TRPV2, which is associated with tumorigenesis in EC, creates an avenue for dual-targeted therapy. We exploited the dual-targeting antitumor efficacy against EC.

**Methods:**

We isolated antigen-specific TCRs (asTCRs) from a naive library constructed with TCRs obtained from enriched cytotoxic T lymphocytes. The robustness of our asTCRs and their TCR-T cell derivatives, Tranilast (TRPV2 inhibitor), and their bivalent treatment were evaluated with prospective cross-reactive human-peptide variants and tumor cells.

**Results:**

Our study demonstrated that our naive unenhanced asTCRs and their TCR-Ts perpetuated their cognate HLA-A*02:01/NY-ESO-1_(157–165)_ specificity, killing varying EC cells with higher cytotoxicity compared to the known affinity-enhanced TCR (TCRe) and its wild-type (TCR0) which targets the same NY-ESO-1 antigen. Furthermore, the TCR-Ts and Tranilast bivalent treatment showed superior EC killing compared to any of their monovalent treatments of either TCR-T or Tranilast.

**Conclusion:**

Our findings suggest that dual-targeted immunotherapy may have a superior antitumor effect. Our study presents a technique to evolve novel, robust, timely therapeutic strategies and interventions for EC and other malignancies.

**Supplementary Information:**

The online version contains supplementary material available at 10.1186/s12935-024-03249-w.

## Introduction and background

Esophageal cancer (EC) is a type of epithelial cancer characterized by NY-ESO-1 expression with an increasing re-expression level in EC tissues, making the usage of autologous or allogeneic T cells engineered to express TCRs that target NY-ESO-1 is a prominent treatment choice [1]. NY-ESO-1 is a cancer/testis antigen that re-expresses in several kinds of malignancy [2]. Its capacity to independently trigger either humoral or cellular inflammatory response, with a restricted expression and a concomitant re-expression in epithelial cancers such as EC [3–5], renders it a suitable target for immunotherapy. Many studies have shown the quintessential tumorigenesis role of NY-ESO-1 antigen via its conserved Pcc-1 domain [6–8]. Its expression across several tumors correlates with the numerous characteristics of tumorigenesis, such as metastasis [3, 9]. It has been reported that the intensity of an antibody-mediated immunity specific to NY-ESO-1 increases as the disease progresses and decreases as the condition regresses [1]. Earlier findings reported associated the stage of EC to NY-ESO-1 detection rate. Locomoting lymphocytes specific for NY-ESO-1 have been linked to improved prognosis, increasing patients’ survival rates. Its ubiquitous expression in several tumor forms and its constricted expression in healthy tissues give it negligible off-target toxicity. Moreover, the high immunogenicity of this tumor-associated antigen (TAA) [6] implies that there may be a chance to strengthen the body’s defenses against it. Its main characteristics reside in its ability to cause cancer patients to develop spontaneous T-cell and antibody responses. Till now, there are several clinical trials at varying phases utilizing a variety of immune-derived treatments to target NY-ESO-1 (http://www.clinicaltrials.gov), including adoptive cell therapies (Additional file 1: Table S4). However, there is yet to be any antigen-specific TCR (asTCR) targeted therapy against EC, although there have been some studies on EC using tumor-infiltrating lymphocytes (TILs). The usage of TIL, however, is limited to antigenic-unspecificity; thus, since the antigen the T cells target is unknown, treatment of EC may not be certain, especially when the antigen profile of the cancer cells or tumor tissues is characterized with heterogeneity. Therefore, utilizing adoptive therapy, antigen-specific TCR-T therapy targeting a prevalent antigen is crucial for improving EC treatment.

EC is hallmarked by the abnormal proliferation of cells in the esophagus. It mostly starts from the epithelium and later metastasizes to other parts of the esophagus. EC is globally common and ranks in the top ten regarding incidence and mortality rates. EC is life-threatening because it evades diagnoses until significant symptoms have appeared, and by that time, the cancer has already reached a relatively advanced stage, leading to a lower survival rate [10]. ECs are conventionally treated by esophagectomy or combined with other treatments [11, 12]. This complex surgery often results in severe complications compounded with recurrence and metastasis. The two types of EC include esophageal squamous cell carcinoma (ESCC) and esophageal adenocarcinoma (EADC). ESCC develops from the epithelial cells lining the esophagus [13], whereas EADC originates in glandular cells [14]. While ESCC accounts for most cases of EC globally, especially in Asia [15, 16], EADC is predominant in Western countries [11]. Currently, EC is amongst the top six cancer-associated mortality [17]. Esophagectomy, after chemo/radiotherapy, has been the remedy for locally advanced ESCC. Several retrospective studies have demonstrated that postoperative radiotherapy could improve the prognosis of patients [18]. Nevertheless, most patients are still developing local–regional relapse and hematological metastasis [18]. These detrimental effects also arise in treating EADC patients. Since NY-ESO-1 is proven to be a prevailing marker in the antigenic profile of EC tumors, it is vital to target NY-ESO-1 on EC using TCR-T therapy. This study reports on the isolation of NY-ESO-1 asTCRs and their corresponding EC antitumor response efficacy in vitro.

Combinatorial therapies have recently improved various immunotherapy interventions [19, 20]. Several studies, including ongoing and completed clinical trials that combined two or more antitumor therapies, yielded superior results over each monovalent treatment [20, 21]. In our previous study, we reported on the antitumor inhibition impact of Tranilast (MK-341) on EC by targeting TRPV2 via the cation-channel pathway [16]. Although Tranilast, developed initially as an anti-allergic agent, has also been utilized in treating autoimmune disorders, etcetera [22], and its tumor inhibition effect on EC complimented with its impotent antiproliferative effect on CD8+ T lymphocytes [23] makes it a suitable candidate to enroll in a drug (Tranilast) and TCR-T combinatorial therapy. This study demonstrated the superiority of Tranilast and TCR-T combinatorial therapy on EC. Our result indicated that EC, when treated with Tranilast combined with TCR-T, yielded significant cytotoxicity against EC compared to Tranilast or TCR-T treatments.

## Materials and methods

### Cell lines

293T (RRID:CVCL_0063), J.RT3-T3.5 (RRID:CVCL_1316), T2 (RRID:CVCL_2211), ESCC cell lines [(TE1 (RRID:CVCL_1759), COLO680N (RRID:CVCL_1131), KYSE30 (RRID:CVCL_1351), ECA109 (RRID:CVCL_6898), KYSE30 (RRID:CVCL_1351), and ECA109 (RRID:CVCL_6898)], and OE19 (RRID:CVCL_1622) EADC cell line were obtained from our institutes’ cell bank. All cell lines used in this study are routinely authenticated and checked for mycoplasma contamination. ECA109 (HLA‐A*02:01^+^) ESCC cell line was stably generated in-house by lentiviral transduction at MOI 5 with HLA‐A*02:01^+^ and puromycin selective marker. Puromycin-resistant cells were selected using 3 μg/ml puromycin (Gibco, cat# A1113803) (Additional file 1: Fig. S6). All the cell lines utilized in this research were maintained in RPMI 1640 medium (Gibco, cat# C11875500BT) augmented with 10% FBS (Gibco, cat# 10099141C). However, 293T cells were grown in DMEM medium (Gibco, cat# C11995500BT) augmented with 10% FBS. NY-ESO-1 expression profile was evaluated with PCR utilizing an amplifiable primer pair (forward: GGCTTCAGGGCTGAATGGAT and reverse: TGAGCCAAAAACACGGGCAG) that covers the epitope of interest. Cells that expressed HLA-A*02:01 were estimated making use of cytometry (BD Accuri C6 flow cytometer, BD Biosciences) after being stained with APC-HLA-A2 antibody (clone BB7.2, BD Biosciences).

### T lymphocytes selection

Peripheral blood mononuclear cells (PBMCs) of three healthy donors were procured from Miaoshun Biotechnology Co., Ltd (Shanghai) (Additional file 1: Table S1). Human T lymphocytes were negatively selected from rested PBMCs using human T lymphocyte isolation kits [STEMCELL Technologies, (CD8+ T lymphocytes, cat# 17953; T lymphocytes, cat# 17951)].

### RNA extraction and cDNA syntheses

All RNA in this study was extracted with TRIzol reagent (Invitrogen, cat# 15596026). Total RNA was extracted from CD8+ cytotoxic T lymphocytes for cDNA synthesis. According to the manufacturer’s manual, all cDNA synthesis in this study was done with PrimeScript IV cDNA Synthesis kit (TaKaRa, cat# 6215A). RNA purity was evaluated at absorbance 260/280 nm using a Nanodrop spectrophotometer (N60/N50 Nanophotometer, IMPLEN), and its integrity was assessed using 1% agarose gel electrophoresis. The quality of synthesized cDNA was evaluated with 1% agarose gel electrophoresis after PCR amplification using various housekeeping genes oligonucleotide (oligo) pairs (Table 1).

**Table 1:** Primer pairs used to assess the quality of synthesized cDNA

Housekeeping gene	Oligo 1 (5ʹ to 3ʹ)	Oligo 2 (5ʹ to 3ʹ)	Product length (bp)
ACTB	GGCTGTGCTATCCCTGTACG	CTTGATCTTCATTGTGCTGGGTG	574
B2M	GCTCGCGCTACTCTCTCTTT	CACGGCAGGCATACTCATCT	278
GAPDH	GACCACAGTCCATGCCATCA	GTCAAAGGTGGAGGAGTGGG	364

PCR amplification oligos for actin beta (ACTB), glyceraldehyde 3-phosphate dehydrogenase (GAPDH), and beta-2-microglobulin (B2M) utilizing cDNA as template

### TCR cDNA library

Degenerate oligonucleotides (Additional file 1: Table S2) were designed, modified, and used to amplify all known αβTCR variable domain genes sourced from the International Immunogenetics (IMGT) Information System server (https://www.imgt.org/), taking cues from previously described methods [24, 25]. All oligonucleotides in this study were synthesized by Sangon Biotech (China). All DNA amplification in this study was set at 30 cycles (98 °C, 30 s; 60 °C, 30 s, and 72 °C, 30 s/kb) following an inceptive denaturation at 98 °C for 2 min using PrimeSTAR Max DNA Polymerase (TaKaRa, cat# R045A). The αβTCR variable domain genes were cloned into a pSCTR-amp phagemid using NcoI/NotI restriction sites and then transformed into TG1 electrocompetent cells. The library was subjected to next-generation sequencing (NGS) and analysis.

### Selection and synthesis of peptides

Relevant and irrelevant peptides (Additional file 1: Table S3) were purchased from Genscript (China). Peptide epitopes were selected based on their immunogenicity and other variables from tumor-associated antigens against specific HLA-allotypes using he Immune Epitope Database (IEDB) server (https://iedb.org/).

### Refolding and purification of peptide human leucocyte antigen

Peptide human leukocyte antigen (pHLA) complexes were purified as previously described [26]. All relevant and irrelevant peptides were refolded from relevant HLA-specific inclusion bodies. Refolded pHLA complexes were purified and biotinylated using ion exchange chromatography (HiTrap QHP column) and size exclusive chromatography (Superdex 75 10/300 GL column) consecutively using AKTA Pure system (GE Healthcare). 10 μl of selected fractions were used for SDS-PAGE analysis to assess the purity of the purified soluble pHLA protein.

### Screening and selection of TCR binders

To screen for TCRs that specifically bind to HLA-A*02:01/NY-ESO-1_(157–165)_ antigenic epitope in a biotinylated pHLA form, three rounds of phage display biopanning was conducted as previously expounded [27]. The ratio of phage recovery (output) against 1 × 10^11^ phage input was titered. Briefly, the biotinylated HLA-A*02:01/NY-ESO-1_(157–165)_ pHLA was captured on streptavidin-coated beads, and then the immobilized pHLA was bound to the displayed TCRs. Non-binding phage was sequentially washed off, whereas bound phage was eluted through trypsinization. The eluents were used to infect TG1 competent cells and cultured overnight at 37 °C on TYE agar augmented with 2% glucose and 100 µg/ml ampicillin. After the third round of panning, single colonies were randomly selected for further screening. Single colonies were inoculated in 2 × TY medium augmented with 100 µg/ml ampicillin and 50 µg/ml kanamycin and displayed for 12–16 h at 30 °C, 220 rpm after being infected with M13 helper phage at a multiplicity of infection (MOI) 1. The TCR-phages were harvested and subjected to monoclonal enzyme-linked immunosorbent assay (ELISA) to affirm their binding to the pHLA.

### Confirmation and identification of antigen-specific TCRs

Single colonies that bound to HLA-A*02:01/NY-ESO-1_(157–165)_ pHLA were assessed for specificity against irrelevant peptides of the same or different antigens.

Biotinylated pHLA (10 µg/ml) was captured at 37 °C for 1 h on pre-blocked 96-well plates coated with 10 µg/ml streptavidin (APExBIO, cat# 9013-20-1). TCR phages were suspended with a blocking buffer (3% mPBS, skimmed milk dissolved in PBS). 100 µl of phage was added into the pHLA-captured wells before incubating for 1 h at 37 °C. 100 μl of M13 HRP-conjugated M13 antibody (SinoBiological, cat# 11973-MM05T-H) was added into the wells at 1:4000 antibody blocking buffer ratio before incubating at 37 °C for 1 h with the binding signals colorimetrically measured at OD450 nm (using Multiskan GO spectrophotometer, ThermoScientific) after adding 100 μl TMB substrate (Beyotime, cat# P0209) for 10 min. Stringent washing with 0.05% PBST (tween 20 suspension in PBS) was carried out after each step. Any TCR that bound to only HLA-A*02:01/NY-ESO-1_(157–165)_ pHLA was identified as asTCRs. asTCR colonies were sanger-sequenced (Sangon Biotech).

### Lentivector construction, lentivirus production, and titration

This study utilized a third-generation packaging system consisting of two packaging lentivectors [(pMDLg/pRRE, Addgene #12251), and (pRSV-Rev, Addgene #12252)], pMD2.G (Addgene #12259) envelope plasmid, and pLTV-mC transfer plasmid under the direction of EF-1α promoter. The transgene(s) were cloned into the transfer plasmid and transformed into Stbl3 competent cells, cultured for 16 h at 37 °C, 220 rpm in LB medium augmented with 100 µg/ml ampicillin. The bacteria culture was pelleted and subjected to plasmid extraction and endotoxin treatment using the EZNA endo-free plasmid maxi kit (OMEGA-Biotek, cat# D6926-03). 293T cells pre-seeded 24 h earlier with 7 × 10^6^ in 100 mm plate were subjected to polyethyleneimine-mediated transfection comprising 2 μg each of the packaging vectors and 6 μg of the transfer vector mixed with Opti-MEM medium (Gibco, cat# 31985-070) were grown at 37 °C, 5% CO_2_ for 8–10 h. The medium was substituted with DMEM containing 10% FBS and further incubated in the same conditions. Lentiviral supernatant was harvested at 48 h and 72 h periods and concentrated with 50 kDa centrifugal concentrators (SARTORIUS, cat# VS04T31). 1.5 × 10^4^ J.RT3-T3.5 cells transduced with serially-diluted virus and cultured for 72 h were cytometrically examined to titer the viruses.

### TCR lentivirus packaging

Sequences of our asTCRs were human-codon optimized and reconstituted into the native TCR heterodimer structure. The alpha and beta chains were separated with a 2A cleavage peptide (Fig. 4a). As described above, concentrated asTCRs were titered after staining transduced J.RT3-T3.5 cells with APC—TCRβ (clone H57-597, BD Biosciences).

### TCR-T manufacturing, activation, and expansion

Activation of T lymphocytes is a prerequisite for expansion in vitro. Briefly, 2 × 10^5^ T cells were actuated making use of CD3/CD28 beads (ThermoFisher, cat# 11131D) in RPMI 1640 medium augmented with 10% FBS and 100 IU/ml IL-2 (Sihuan Shengwu, Lot# S10970016) to generate co-stimulatory signals that co-achieve antigen-induced activation with TCR. Stimulated T cells were treated with 10 µg/ml protamine sulfate (Macklin, cat# P913917) and then transduced with asTCR-lentiviruses at MOI 10 to produce the TCR-Ts. The TCR-Ts were expanded and used for functional assays and analysis. Before doing functional assays, expanded TCR-Ts were quantified and evaluated for expressed TCR and activation markers (Fig. 5a).

### TCR-T functional assays

The function of our TCR-Ts was evaluated through a specific-killing (cytotoxicity) assay and the activation and release of interferon-gamma (IFNγ) by T cells upon encountering an antigen.

TCR-T-mediated cytotoxicity was evaluated with CytoTox 96 kit (Promega, cat# G1780) by measuring the release of lactate dehydrogenase (LDH) by damaged cancer cells. Briefly, 2 × 10^4^ cancer cells (target, T) expressing or not expressing HLA-A*2:01/NY-ESO-1_(157–165)_ were co-cultured overnight with TCR-Ts (effector, E) of varying effector-to-target (E:T) at 37 °C, 5% CO_2_. The cells were spun down, and 50 µl CytoTox 96 solution was added to 50 µl cell supernatant in a fresh 96-well plate and left to stand at 25 °C for 0.5 h, then 50 µl stop solution was added. LDH release was colorimetrically quantified at OD490 nm using Multiskan GO spectrophotometer (ThermoScientific). Cytotoxicity percentage was calculated after standardization using the formula below:

$${\text{Cytotoxicity }}\left( \% \right) = \frac{{\left( {{\text{experimental}}{-}{\text{only effector cells}}{-}{\text{only target cells}}} \right) \times 100}}{{{\text{lysed target cells}}{-}{\text{only target cells}}}}$$

Additionally, the TCR-Ts effector function was analyzed via antigen recognition and activation by estimating the amount of IFNγ released. Following the manufacturer’s instruction, IFNγ release was measured using a human IFNγ sandwich ELISA kit (SinoBiological, cat# KIT11725A). Briefly, TCR-Ts with the same E:T ratios described above were either co-cultured with cancer cells or T2 cells pulsed with various antigen peptides. IFNγ in cell culture supernatant was used to develop ELISA by binding to the immobilized antibody in an IFNγ pre-coated plate. Unbound substances were washed off; after that, IFNγ detection antibody was added to bind to the capture antibody and IFNγ in the sample. IFNγ release was then colorimetrically quantified at OD450 nm (using Multiskan GO spectrophotometer, ThermoScientific) following the addition of a colorimetric conjugated enzyme and a stop solution.

### Tranilast or TCR-T and Tranilast combinatorial-mediated cytotoxicity assay

Tranilast or TCR-T and Tranilast combinatorial-mediated cytotoxicity assay were examined using cell counting kit—8 (CCK-8) (Biosharp, cat# BS350B). Briefly, 2 × 10^4^ cancer cells were cultured or co-cultured with TCR-Ts of different E:T overnight at 37 °C, 5% CO_2_ in 120 μM Tranilast (TargetMol, cat# T2690). The EC50 value of Tranilast was confirmed from a drug-dosage response assay curve after adding CCK-8 solution was added to each well. CCK-8 utilized tetrazolium salt reduced by cell dehydrogenases to give a yellow-colored product (formazan). The amount of formazan generated by dehydrogenases corresponded to viable cells colorimetrically estimated at OD450 nm using Multiskan GO spectrophotometer (ThermoScientific). The rate of target cell inhibition was calculated using the formula below:

$${\text{Inhibition rate}}\left( \% \right) = \frac{{\left( {{\text{Control well absorbance}} - {\text{Experimental well absorbance}}} \right){ } \times { }100}}{{{\text{Control well absorbance}} - {\text{Blank well absorbance}}}}$$

### Statistical analyses

All statistical analyses were conducted with GraphPad Prism 9. Data are shown as mean ± SD and compared using one-way ANOVA (Brown-Forsythe and Welch tests) with p < 0.05 considered significant. Dose–response curve analysis was done using nonlinear regression interpolated at 95% confidence interval. The NGS data (identity match) was analyzed using Python 3.8. All the data in this research is contained in the main article and its supplementary materials.

## Results

### Cytotoxic T lymphocyte receptor library is highly diversified

**Fig. 1:**
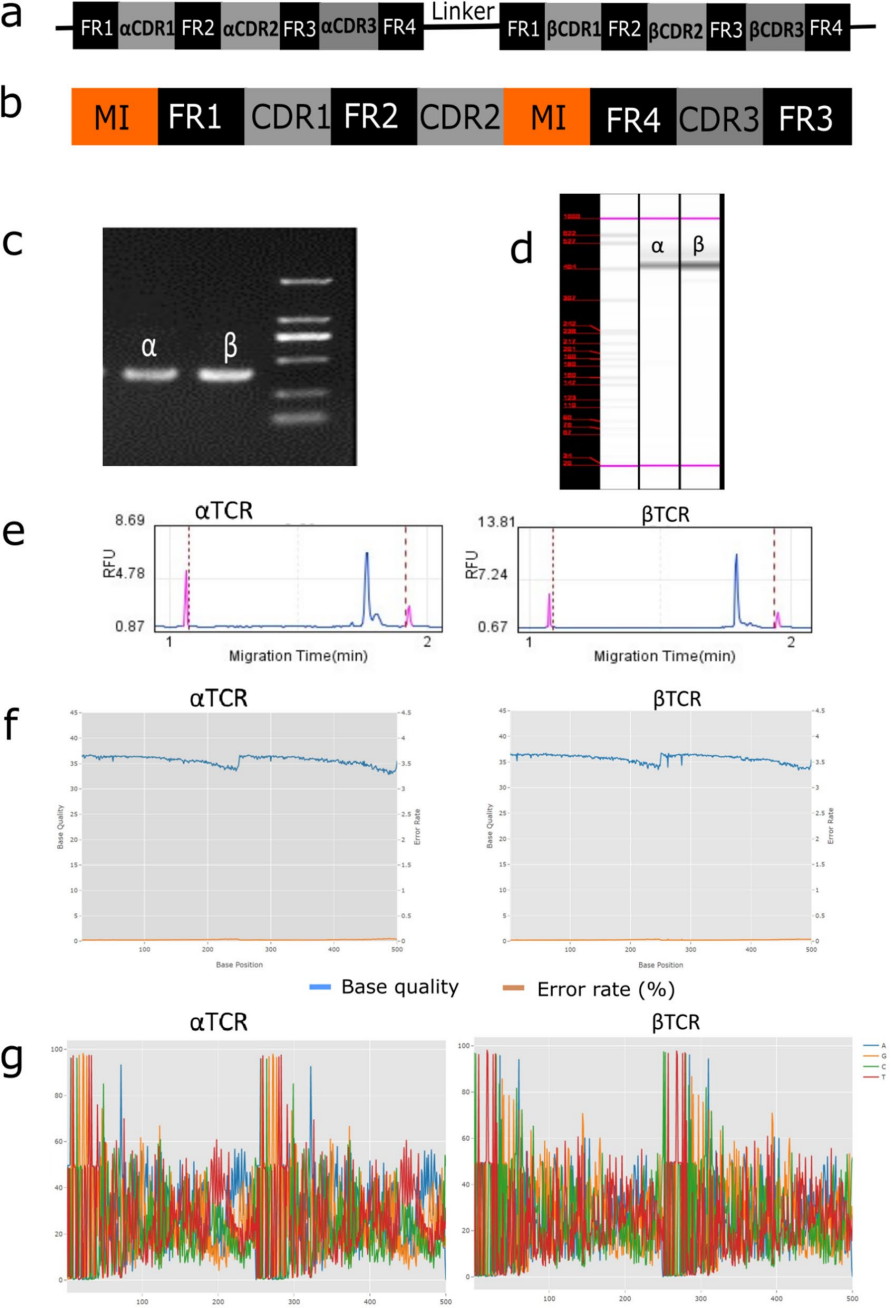
NGS-quality assessment of αβTCR library. **a** schematic representation of the original gene arrangement of αβTCR variable chain. A linker separates αTCR and βTCR. **b** schematic representation of separate αTCR or βTCR indexed with molecular identifiers. **c**–**f** TCR DNA quality and size characterization. Fragmented αTCR and βTCR Trap DNA of about 480 bp (**c**). αTCR and βTCR DNA smear integrity data (**d**). Electropherogram diagram showing the size distribution range of αTCR and βTCR DNA libraries as migration time versus intensity (relative fluorescence unit, RFU) spectrum (**e**). Evaluation of the quality of bases read for each αTCR and βTCR library (**f**). **g** diversity of αTCR and βTCR libraries. Base numbers 0 to 55 and 251 to 300 represent molecular identifiers, base numbers 56 to 250 and 301 to 500 represent the variable (V) region of the αTCR and βTCR

The TCR library was subjected to next-generation sequencing (NGS) and analysis to evaluate its quality (Fig. 1). The library was PCR-enriched into αTCR and βTCR, fragmented into size-ranging DNA compatible for Illumina, and indexed with molecular identifiers to enable multiplexing (Fig. 1a–f). Each DNA library was evaluated using bioanalyzer systems to determine the integrity and base quality. NGS analysis showed a high diversity at the TCR complementarity determining region (CDR) of both αTCR and βTCR libraries with well-balanced or roughly distributed nucleotides at the variable domains, whereas the molecular identifier regions showed lower diversity with unbalanced nucleotide distribution (Fig. 1g).

**Fig. 2:**
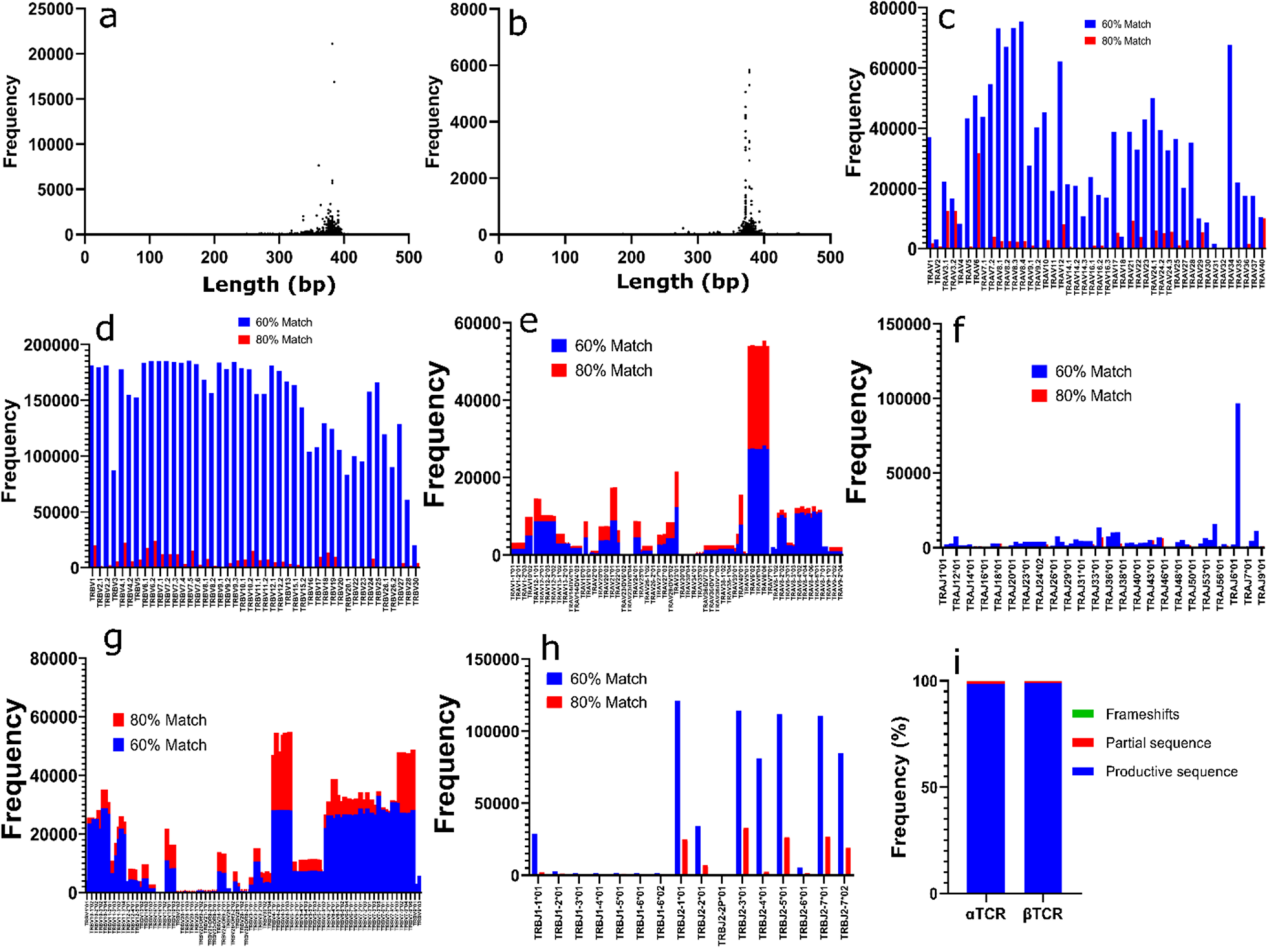
Identity characterization of unique TCR clones. **a**, **b** dot plots showing the size frequency of each unique αTCR and βTCR, respectively. **c**, **d** frequency of all TRAV and TRBV oligonucleotides distributed across each αTCR and βTCR oligonucleotides respective library. **e**–**h**, **f**, frequency distribution of all; αTCR V and J genes (**e** and **f**), and βTCR V and J genes (**g** and **h**). **i** the libraries’ frequency distribution of all TCR productive sequences, partial sequences, and frameshifts

All known αβTCR genes and their degenerate oligonucleotides used in building the libraries were identified and matched to evaluate the frequencies of specific gene distributions. All the TCR genes averaged a size of about 400 bp (Fig. 2a and b). αβTCR variable domains mainly comprise the variable (V) and junction (J) genes. Match scores were assigned to specific degenerate oligonucleotides (Fig. 2c and d) and specific TCR genes (Fig. 2e–h) by 60% or 80% identity. The number of productive genes, partial sequences, and frameshifts were also estimated (Fig. 2i).

### ***Biopanning and characterization of NY-ESO-1***_***(157–165)***_*** TCR binders***

**Fig. 3:**
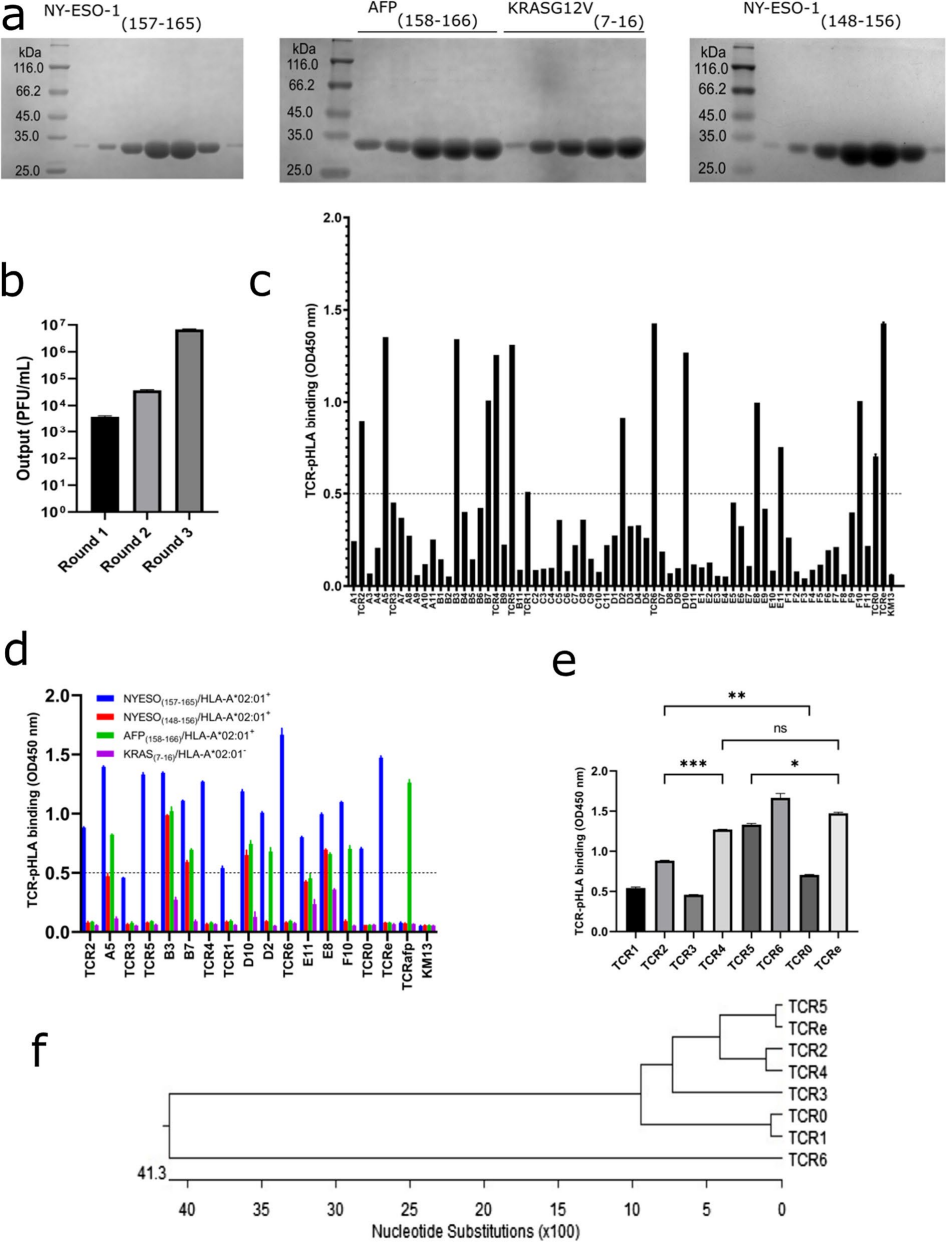
Screening of asTCRs. **a** Collected fractions of size-exclusive chromatography-purified soluble pHLAs are presented on 12% non-reducing SDS-PAGE. **b** TCR-phage output obtained from three rounds of panning against NY-ESO-1_(157–165)_ pHLA. **c** Monoclonal ELISA of 66 TCR clones randomly selected to evaluate their binding with NY-ESO-1_(157–165)_ pHLA. **d** Monoclonal ELISA of NY-ESO-1_(157–165)_ pHLA-TCR binders against irrelevant pHLA. asTCRs were identified when they only bound to _(157–165)_ pHLA. **e** asTCRs binding variance. All ELISA values at OD450 nm were normalized against signal from KM13 with TCRafp, an AFP_(158–166)_ asTCR serving as a control standard. **f** asTCRs phylogenetic family. TCR0 and TCRe represent 1G4 TCR wild-type and its affinity-enhanced TCR derived from the directed evolution of its CDRS. TCR1, TCR2, TCR3, TCR4, TCR5, and TCR6 are asTCRs selected from the naïve library after normalization. Most asTCRs were selected around the threshold of OD450 nm = 0.5. Data are arrayed as mean ± SD and compared making use of one-way ANOVA (Brown-Forsythe and Welch multiple comparison tests) where *p < 0.05, **p < 0.01, ***p < 0.001, ****p < 0.0001

Three rounds of biopanning were performed to screen TCRs that bind to NY-ESO-1_(157–165)_ pHLA. Purified soluble pHLAs were used for panning and affinity binding assays (Fig. 3a, and Additional file 1: Table S3). As shown in Fig. 3b, phage output in each consecutive round increased, which is typical of an efficient screening. Following the third pan, 66 clones were randomly selected for ELISA, and 23 clones were identified as good binders after normalization at a threshold of OD450 nm = 0.5 (Fig. 3c). The 23 selected binders were subjected to antigen-specificity assay against other irrelevant pHLA; 6 TCRs bound to only NY-ESO-1_(157–165)_ (Fig. 3d) and were identified as asTCRs_._ The binding differences amongst the asTCRs were also estimated (Fig. 3e), and their sequences were analyzed for gene identification and phylogeny (Table 2, Fig. 3f, and Additional file 1: Fig. S2).

**Table 2:** Synopsis of selected as TCRs’ gene identity and characterization

TCR ID	Binding affinity, K_d_ (μM)	Closest αTCR V-gene	Identity (%)	Closest αTCR J-gene	Identity (%)	Closest βTCR V-gene	Identity (%)	Closest βTCR J-gene	Identity (%)
TCR0	32	Homsap TRAV21*01 F	70.74	Homsap TRAJ6*01 F	72.58	Homsap TRBV6-5*01 F	78.02	Homsap TRBJ2-2*01 F	82.00
TCR1	NE	Homsap TRAV21*01 F	70.74	Homsap TRAJ6*01 F	72.58	Homsap TRBV6-8*01 F	72.96	Homsap TRBJ2-7*01 F	71.43
TCR2	NE	Homsap TRAV21*02 (F)	74.81	Homsap TRAJ6*01 F	64.52	Homsap TRBV6-6*04 (F)	72.89	Homsap TRBJ2-2*01 F	73.91
TCR3	NE	Homsap TRAV21*01 F	74.44	Homsap TRAJ6*01 F	64.52	Homsap TRBV6-5*01 F	97.44	Homsap TRBJ2-3*01 F	83.33
TCR4	NE	Homsap TRAV21*02 (F)	74.81	Homsap TRAJ6*01 F	69.35	Homsap TRBV6-8*01 F	73.33	Homsap TRBJ2-2*01 F	82.00
TCR5	NE	Homsap TRAV21*02 (F)	74.81	Homsap TRAJ6*01 F	69.35	Homsap TRBV6-6*03 (F)	72.53	Homsap TRBJ2-2*01 F	80.00
TCR6	NE	Homsap TRAV17*01 F	100.00	Homsap TRAJ40*01 F	92.98	Homsap TRBV5-4*02 (F)	99.63	Homsap TRBJ1-1*01 F	87.50
TCRe	1.07	Homsap TRAV21*01 F	74.44	Homsap TRAJ6*01 F	64.52	Homsap TRBV6-5*01 F	76.92	Homsap TRBJ2-2*01 F	80.00

The closest αβTCR V and J genes phylogeny was analyzed from the IMGT server. Kds were previously [28, 29] estimated using Biacore surface plasmon resonance with soluble TCRs and NY-ESO-1_(157–165)_ pHLA. *NE* not estimated

### TCR-Ts production and expansion

**Fig. 4:**
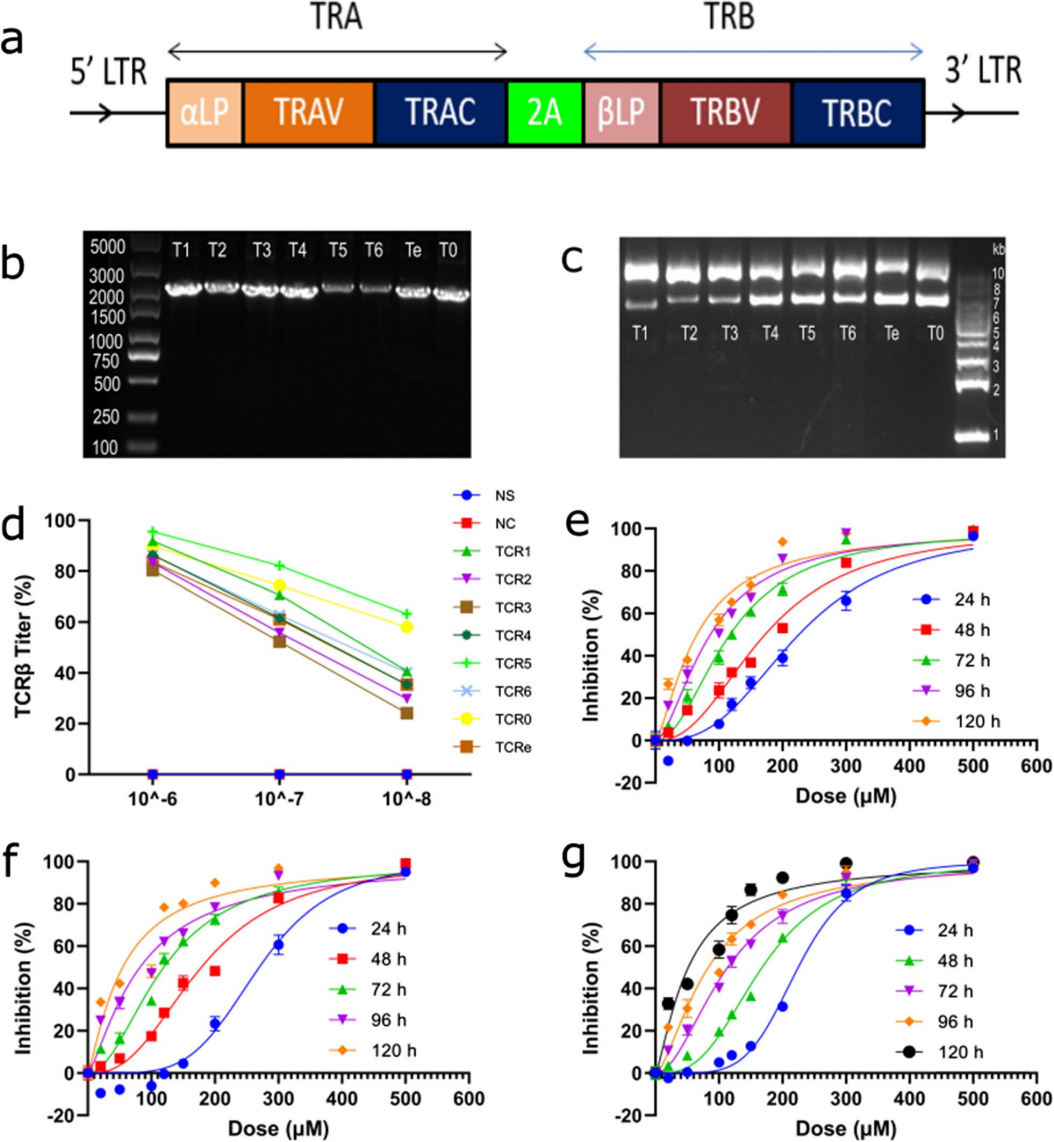
asTCR lentivirus production and Tranilast dose–response curve. **a** Framework of αβTCR transgene. The αTCR and βTCR consisting of the leader peptide, variable, and constant genes were separated by a 2A cleavage peptide. **b** and **c** amplified asTCR transgenes of about 2.2 kb size and their respective dimeric lentivector clones. Lanes T1 to T0 represent TCR1 to TCR0, respectively. **d** Titration of asTCR lentiviruses after transducing J.RT3-T3.5 cells. Lentivirus was titered using TCRβ antibody in a flow cytometry analysis. **e**–**g** Hillslope representing the diagrammatic representation of Tranilast dose–response relationship on ECA109(NY-ESO-1^−^/HLA-A*02:01^−^) (**e**), ECA109(NY-ESO-1^+^/HLA-A*02:01^+^) (**f**), and OE19(NY-ESO-1^+^/HLA-A*02:01^+^) (**g**) EC cell lines

**Fig. 5:**
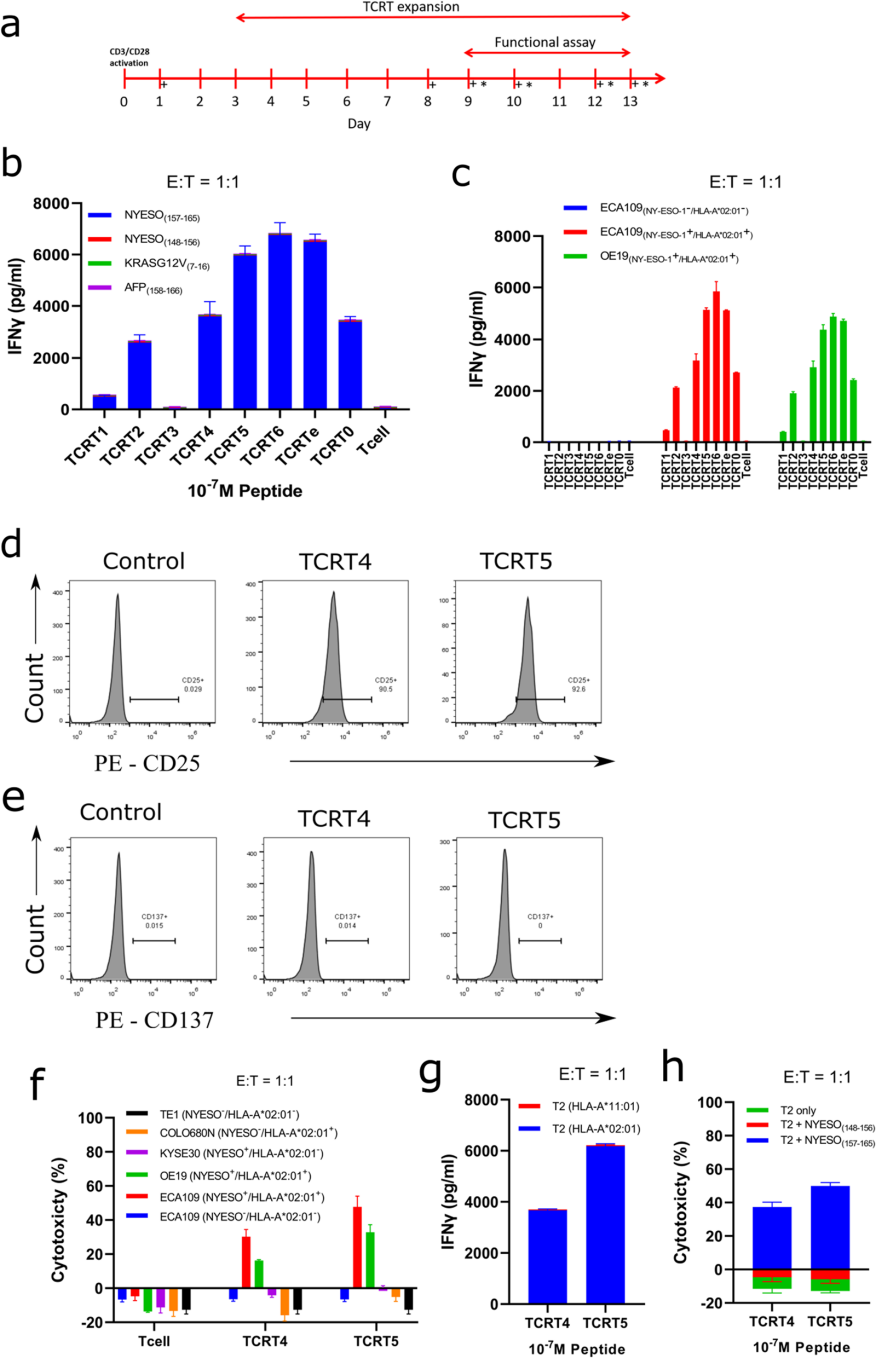
TCR-T production and functional assay. **a** Framework of TCR-T production, activation, and expansion. T cells were activated on day 0, transduced with asTCR lentivirus on day 1, and expanded for two weeks. (+) sign indicates days for evaluating and quantifying essential T cell markers, and (*) asterisks indicate days for performing functional assays. **b** IFNγ was detected after co-culturing TCR-Ts with (NY-ESO-1(_157–165_)) and other peptides-enriched T2 cells to validate TCR-T function and specificity. **c** TCR-Ts activation and potential antitumor function were evaluated by quantifying IFNγ release after an overnight co-culturing of TCR-Ts with ECA109(NY-ESO-1^−^/HLA-A*02:01^−^), ECA109(NY-ESO-1^+^/HLA-A*02:01^+^) and OE19(NY-ESO-1^+^/HLA-A*02:01^+^) EC cells. **d** T cell activation before performing functional assay was evaluated using a late T cell activation marker (CD25) by selecting sampling two different TCR-Ts. **e** T cell resting phase was verified before performing functional assay using CD137 marker using two different sampled TCR-Ts. **f**–**h** TCR-T function and specificity of two selected TCR-Ts (TCRT4 and TCRT5) were validated by quantifying their IFNγ release or killing effect (cytotoxicity) against multiple cancer cells with activated native T cell as negative control (**f**), T2 cells expressing different HLA allotype pulsed with relevant peptide (NY-ESO-1_(157–165)_) (**g**), and (NY-ESO-1_(157–165)_) or other peptides-enriched T2 cells (**h**)

**Fig. 6:**
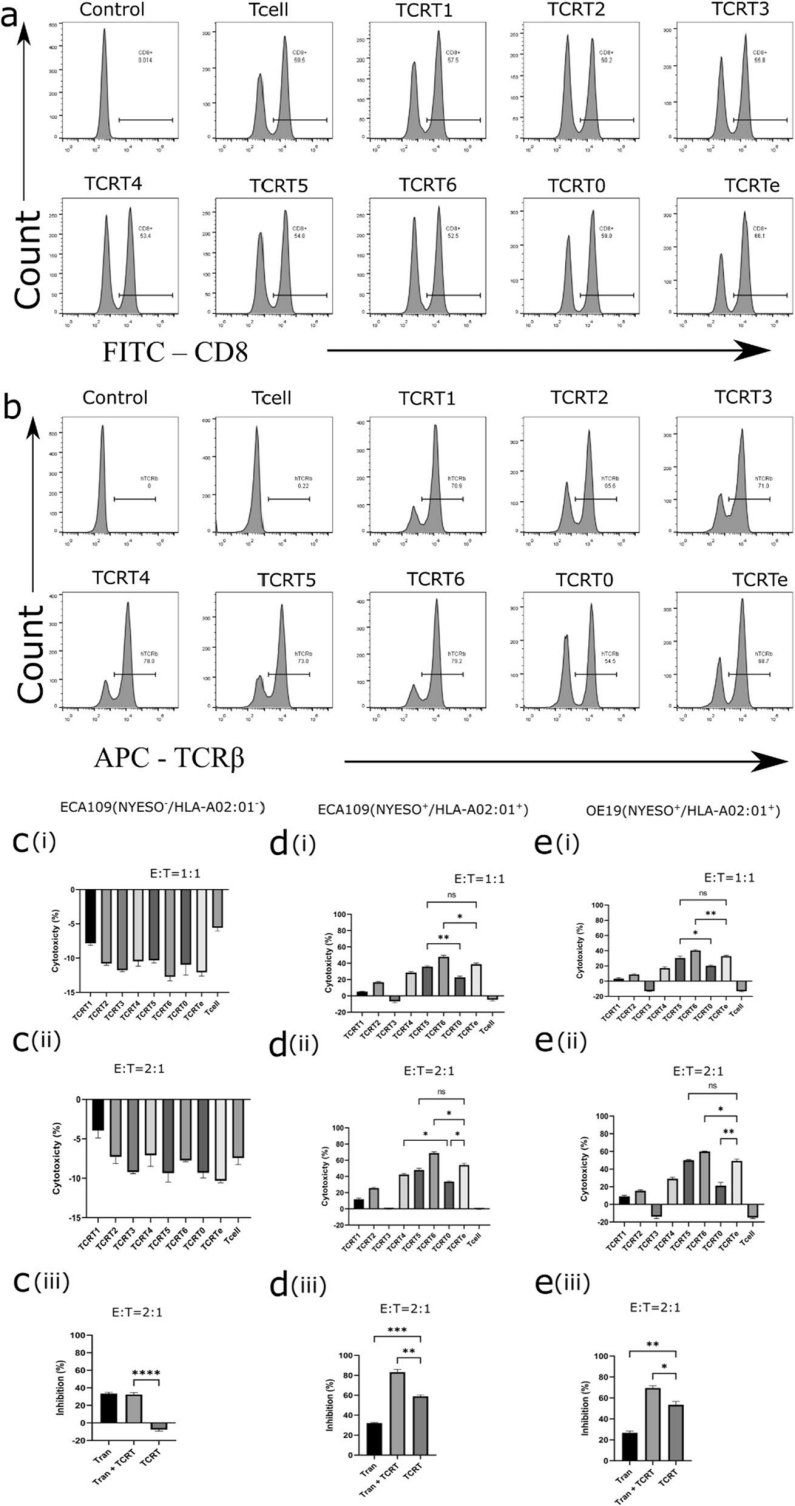
TCR-T-mediated or Tranilast-mediated cytotoxicity. **a** Manufactured and expanded TCR-Ts were evaluated for the quantity of cytotoxic CD8+ lymphocytes via cytometry with unstained cells as a negative control and native T cell population as a positive control. **b** Expanded TCR-Ts were evaluated for the quantity expressing asTCRs via flow cytometry with unstained T cells and native T cells as negative controls. **c** TCR-T or Tranilast killing effect after co-culturing with ECA109(NY-ESO-1^−^/HLA-A*02:01^−^) ESCC cells; cytotoxicity of various TCR-Ts against ECA109(NY-ESO-1^−^/HLA-A*02:01^−^) at E:T = 1:1 (**c(i)**), E:T = 2:1 (**c(ii)**); and the killing effect of Tranilast, selected TCR-T or a combination of both using 120 μM Tranilast or E:T = 1:1 for TCR-Ts and cancer cells (**c(iii)**). **d** TCR-T or Tranilast killing effect after co-culturing with ECA109(NY-ESO-1^+^/HLA-A*02:01^+^) ESCC cells; cytotoxicity of various TCR-Ts against ECA109(NY-ESO-1^+^/HLA-A*02:01^+^) at E:T = 1:1 (**d(i)**), E:T = 2:1 (d**(ii)**); and the killing effect of Tranilast, selected TCR-T or a combination of both using 120 μM Tranilast or E:T = 2:1 for TCR-Ts and cancer cells (**d(iii)**). **e** TCR-T or Tranilast killing effect after co-culturing with OE19(NY-ESO-1^+^/HLA-A*02:01^+^) EADC cells; cytotoxicity of various TCR-Ts against OE19(NY-ESO-1^+^/HLA-A*02:01^+^) at E:T = 1:1 (**e(i)**), E:T = 2:1 (**e(ii)**); and the killing effect of Tranilast, selected TCR-T or a combination of both using 120 μM Tranilast or E:T = 2:1 for TCR-Ts and cancer cells (**e(iii)**). Data are shown as mean ± SD and compared using one-way ANOVA (Brown-Forsythe and Welch tests) with *p < 0.05, **p < 0.01, ***p < 0.001, ****p < 0.0001

To make TCR-Ts, asTCR transgenes were cloned into a lentiviral transfer plasmid (Fig. 4a–c) and used to produce lentivirus. Concentrated lentivirus was titered using TCRβ antibody (Fig. 4d). TCR-Ts were made by transducing activated T cells with asTCR-lentiviruses and expanded for two weeks (Fig. 5a). Prior to performing to evaluating manufactured and expanded TCR-Ts function, surface markers for activated T cells, asTCR, and other essential markers for the effector function of cytotoxic T lymphocytes were quantified using flow cytometry. Our data showed TCR-T populations with up to 66% cytotoxic CD8+ T cells (Fig. 6a), up to 78% asTCR expression with 0% asTCR expression on native T cell population (Fig. 6b), over 90% activation of sampled TCR-Ts (Fig. 5d) and 0% antigen presentation of sampled TCR-Ts (Fig. 5e) indicative of a resting T cell that is yet to encounter an antigen.

### TCR-Ts redirect esophageal cancer-killing

To investigate the effector function of our TCR-Ts against EC, we set up in vitro cytotoxicity and IFNγ release assays where we co-cultured our TCR-Ts with varying cancer cell lines or TCR-Ts with T2 cells pulsed with various antigenic peptides at E:T ratios 1:1 or 2:1. NY-ESO-1 of the EC cell lines were qualitatively verified with PCR (Additional file 1: Fig. S3) and HLA-A*02:01 was quantitatively verified by flow cytometry (Additional file 1: Fig. S4). TCRT2, TCRT4, TCRT5, TCRT6, TCRT0, and TCRTe showed significant levels of killing effect and IFNγ release against EC cell lines compared to the native T cell, with TCRT2 and TCRT6 having the lowest and highest killing effect (Fig. 6c–ei and ii) or IFNγ release (Fig. 5c), respectively, across all E:T ratios. All TCR-Ts showed neither killing effect nor IFNγ release on ECA109(NY-ESO-1^−^/HLA-A*02:01^−^) due to the lack of NY-ESO-1 and HLA-A*02:01 expression. These TCR-Ts had a killing effect only on EC cell lines expressing both HLA-A*02:01 and NY-ESO-1 antigen. However, TCRT1 and TCRT3 had minimal or no killing effect or IFNγ release on EC cell lines across all E:T ratios indicative of inferior function (TCRT1) or no function (TCRT3) consistent with the TCR and specific antigen binding assay (Fig. 3c–e). Our TCR-Ts exhibited up to 45% and 65% lysis against EC cells at various E:T ratios respectively (Fig. 6). Sampled TCR-Ts with good function was validated against a wide range of cells and the result showed specific cytotoxicity against relevant cancer cells expressing both HLA-A*02:01 and NY-ESO-1 antigen (Fig. 5f) and relevant peptide-enriched T2 cells or native T2 cells as negative control (Fig. 5h).

IFNγ release against peptide-enriched T2 cells confirmed the function of our TCR-Ts with no IFNγ detected for irrelevant peptides but with a significant release for the relevant peptide (Fig. 5b). Sampled TCR-Ts with good function were validated against T2 cells expressing different HLA allotype, the result showed that, only relevant-peptide enriched T2 cells expressing HLA-A*02:01 detected IFNγ (Fig. 5g).

### TCR-T and Tranilast bivalent treatment yields superior EC killing over monovalent treatments

**Fig. 7:**
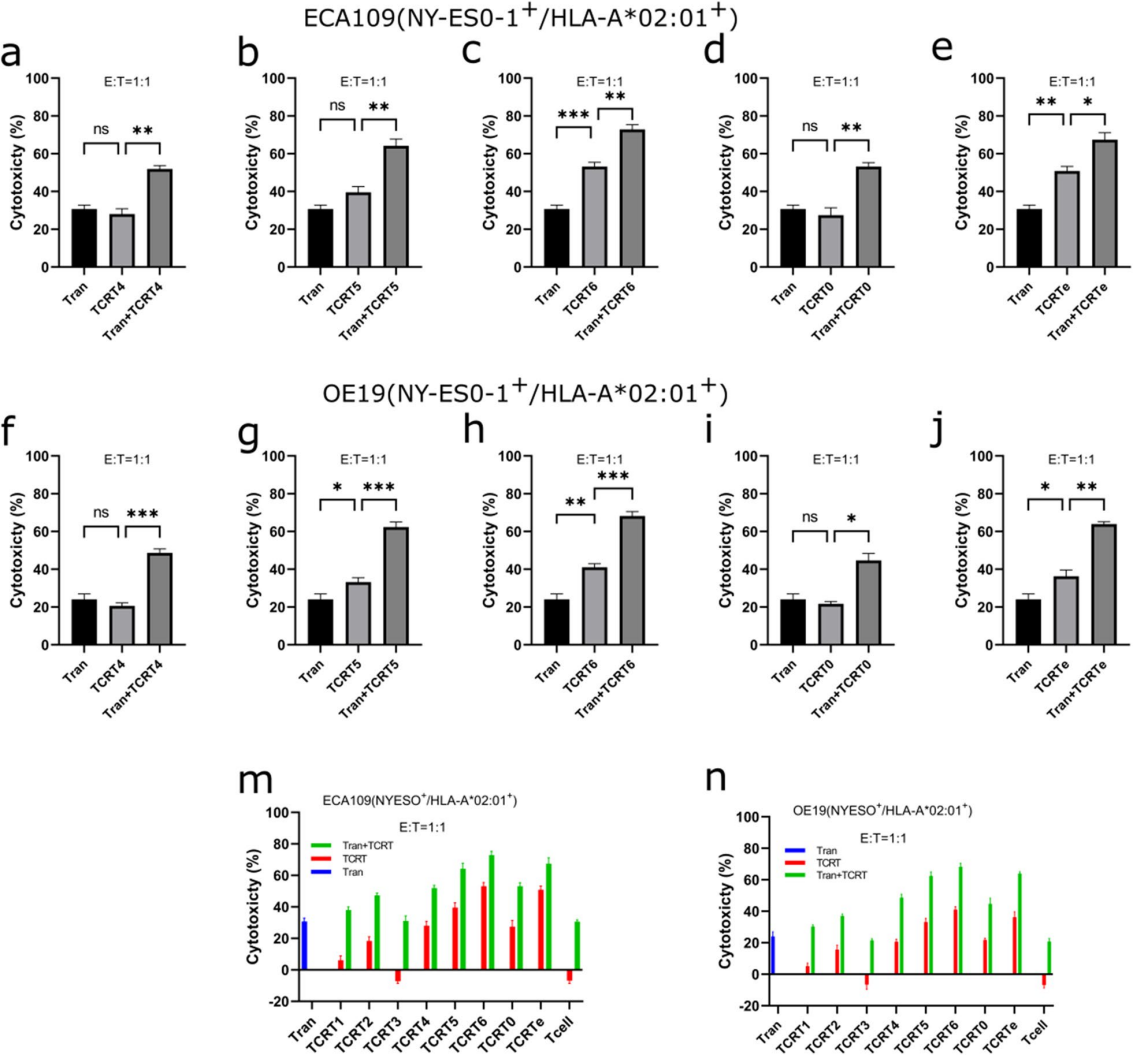
TCR-T and Tranilast bivalent-mediated cytotoxicity. **a**–**e** Monovalent and bivalent treatments of Tranilast and TCR-Ts against ECA109(NY-ESO-1^+^/HLA-A*02:01^+^) ESCC cells. The killing effect of either Tranilast or TCR-T or a combination of both for each selected TCR-T was quantified by LDH release. **f**–**j** Monovalent and bivalent treatments of Tranilast and TCR-Ts against OE19(NY-ESO-1^+^/HLA-A*02:01^+^) EADC cells. The killing effect of either Tranilast or TCR-T or a combination of both for each selected TCR-T was quantified by LDH released by the cancer cells. **m** and **n** monovalent TCR-Ts or Tranilast treatment against ECA109(NY-ESO-1^+^/HLA-A*02:01^+^) cells (**m**) and OE19(NY-ESO-1^+^/HLA-A*02:01^+^) cells (**n**) using native T cells non-functional TCR-T (TCR-T3), and Tranilast as controls. Data are shown as mean ± SD and compared using one-way ANOVA (Brown-Forsythe and Welch tests) with *p < 0.05, **p < 0.01, ***p < 0.001

To evaluate the combinatorial EC killing effect of TCRT and Tranilast, we estimated the EC50 values of Tranilast against varying EC cell lines (Fig. 4e–g, Additional file 1: Table S5). We confirmed the dose (120 μM) of Tranilast needed for effective treatment of EC cell lines as previously reported [16]. We previously demonstrated the anticancer effect of Tranilast against EC by antagonizing TRPV2, which is responsible for promoting EC tumorigenesis via the HSP70/27 and PI3K/Akt/mTOR pathways; the result also showed no off-target cytotoxicity, especially against non-tumor cells and tissues [16]. TCR-T and Tranilast bivalent treatment showed a superior killing effect over the individual monovalent treatments of either Tranilast only or TCR-T only (Fig. 6d(iii) and e(iii)). TCR-Ts and Tranilast bivalent treatment showed up to 85% specific lysis against EC cells (Fig. 6c(iii), d(iii), and e(iii)). All TCR-Ts with good killing efficacy yielded superior killing effects against EC when combined with Tranilast with significant differences compared to only TCR-T or Tranilast treatment (Fig. 7).

## Discussion

TCR recognizes and binds to peptide-antigens displayed by HLA through cancer-antigen presentation, leading to the initiation of an immune response [29, 30]. To fully understand the characteristic nature of the binding of TCR to an antigenic epitope, the repertoire and etiology of the TCR or T cell must be known. The significant diversity of TCR is produced by the randomization and rearrangement of the V-D-J genes of the TCR variable domains [31, 32]; once assembled, the heterodimer TCR gives specificity to naive T cells. The utmost diversity of the TCR repertoire constitutes a significant analytical obstacle prompting several studies concerning the TCR repertoire analysis [31–33]. This study delved into three healthy donors’ combined TCR repertoire analysis (Additional file 1: Table S1). The TCR profile of these donors showed the utmost quality of functional genes and diversity, representing over 95% of known human TCR genes (Fig. 2e–h) as published on the IMGT server. However, the specific gene identity matches were less than 100%, confirming that individuals may have similar gene sets but with microvariations. Our donors showed a highly diversified repertoire but were limited by the lack of specific genes (TRAV8-7*01, TRBV26*01, TRBV26*02, and TRBV26/OR9-2*01). As previously reported, this could result from geographical location, ethnicity, or ancestry [25], confirming that individuals of the same ancestral origin may have different TCR profiles than individuals of distant ancestry. A highly diversified TCR repertoire creates an avenue for identifying and isolating a plethora of TCRs against a wide range of antigens or cancer biomarkers, as previously reported [33, 34]; our library has demonstrated all the characteristics required for a quality library with over 95% functional genes and an insignificant percentage of frameshifts and partial sequences (Fig. 2i). The library was able to select several asTCRs after thoroughly screening it against varying range of antigens (data not shown). Rigorously screening of these TCRs against NY-ESO-1_(157–165)_-SLLMWITQC antigenic epitope resulted in the isolation of asTCRs with relatively similar and also higher-binding affinities compared to the famous 1G4 TCR (TCR0), which is specific to NY-ESO-1_(157–165)_-SLLMWITQC (Fig. 3d, e, Table 2). Conventionally, affinities of TCRs against specific antigens are enhanced through laborious and costly affinity maturation evolution processes that involve codon substitutions of the CDRs of the wild-type TCRs. Our study, however, was able to naturally isolate higher-binding asTCRs comparable to the affinity-enhanced IG4-TCR (TCRe) (Fig. 3e) with corresponding effector function when transduced onto T lymphocytes (Figs. 5b, c, and 6c–e). Our asTCRs, by virtue of their diversity and phylogeny analysis, showed that thoroughly screening naive libraries may yield higher affinity-binding asTCRs with the same or better effector or cytotoxic function, achieving the same result as doing the laborious and costly affinity maturation of the CDRs of a low-affinity wild-type TCR, ultimately providing a robust and timely intervention for adoptive T cell therapy.

This study scrutinized the cancer-killing efficacy of T cells transduced with different asTCRs targeting HLA-A*02:01/NY-ESO-1_(157–165)_ nonapeptide epitope. The binding specificity of these asTCRs was molecularly investigated by ELISA (Fig. 3c–e) against relevant and/or irrelevant peptides refolded into soluble antigens (pHLAs) (Fig. 3a, and Additional file 1: Table S3). The antitumor response efficacy of our TCR-Ts was evaluated against EADC and ESCC cells by quantifying the cytotoxicity and IFNγ release after an encounter with cancer cells or antigens. NY-ESO-1 (Additional file 1: Fig. S3) and HLA-A2 (Additional file 1: Fig. S4) expression profiles of EC cell lines corresponded to the anticipated killing effect of our TCRTs. Though there have been various immunotherapy interventions targeting NY-ESO-1 biomarker at different pre-clinical and clinical trial phases (Additional file 1: Table S4), there is yet to be a study or intervention that targets NY-ESO-1 in EC and, more so, yet to be adoptive T cell therapy targeting the same biomarker. Targeting NY-ESO-1 with TCR-Ts eliminates the imperilment of autoimmunity by virtue of NY-ESO-1 being sturdily expressed by the normal adult testes, spermatogonia, and spermatocytes. TCR-Ts eliminate this threat by only recognizing and binding to NY-ESO-1 in the form of antigen presentation by the HLA complex. That is, in the absence of antigen presentation (which is peculiar to tumor cells), TCRs on the surface of T cells neither recognize nor bind to NY-ESO-1 to initiate an inflammatory response. Also, effortlessly and safely expanding TCR-Ts over a short period is a crucial consideration for immunotherapy. We expanded our TCR-Ts over 600-fold within two weeks after T cell transduction with asTCR lentiviruses (data not shown).

TCR clones A5, B3, B7, D10, E8, and F10 showed higher binding with the relevant pHLA (Fig. 3c); however, these higher binders also bound with irrelevant pHLAs (Fig. 3d), demonstrating cross-reactivity. Cross-reactivity may be good if the TCR also targets other known TAAs, but it becomes a disadvantage when the target of the TCR is unknown. Further studies could delve into the potential benefits of TCR-multiple targeting. Enhancing the affinity of an asTCR could yield a higher cytotoxic effector T cell function against a particular tumor; however, studies have shown some disadvantages that come with it. Zhao et al. reported that affinity-enhanced asTCRs exhibited specific antigen recognition but were limited with cross-reactivity with increasing affinity [35]. That is to say, artificially enhancing the affinity of asTCRs could result in a higher killing efficiency but also coupled with deleterious effects such as unspecific binding either to a different epitope of the same antigen or to a different antigen. A previous report, in the instance of affinity-enhanced asTCRs originating from the wild-type 1G4 asTCR, showed unspecific binding and killing effect against cancer cells that were either HLA-A*02:01 negative or NY-ESO-1 negative, although the wild-type 1G4 asTCR is restricted to NY-ESO-1/HLA-A*02:01 [28, 35]. Our study clearly showed that our naturally affinity-unenhanced asTCRs have intermediate and increased binding and TCR-T effector functions compared to the 1G4 wild-type and its affinity-enhanced asTCR with sturdy specificity (Figs. 2e, and 5c–e). Notably, the cytotoxic effector function of TCRT6 showed significantly better killing activity than the affinity-enhanced TCR (TCRTe). TCRT5 and TCRTe showed relatively similar killing effects. TCRT4, TCRT5, and TCRT6 showed higher cancer-killing effects than the wild-type 1G4 TCR-T (TCRT0). At all E: T ratios, TCRT2 and TCRT0 showed relatively similar cancer-killing effects against ESCC and EADC cell lines. However, TCRT1 showed a weaker or lower killing effect than TCRT0, whereas TCRT3 showed no killing effect as anticipated. TCRT2, TCRT4, TCRT5, TCRT6, TCRT0, and TCRTe only had a killing effect on double positive (HLA-A*2:01^+^/NYE-SO-1^+^) target cells with no killing effect on single positive or double negative target cells (Figs. 5c, 6c). The antitumor function of each TCR-T was confirmed by the detection of IFNγ released (Fig. 5b, c). Before performing cytotoxic assays, important T cell markers such as CD25, a late T cell activation marker, and CD137, which indicates the active or resting phase of antigen-specific T cells, were investigated. Our data showed over 90% expression of CD25 (Fig. 5d) and about 0% expression of CD137 (Fig. 5e) markers of selected TCRTs, indicating an activated TCRT in their resting phase because they were yet to encounter any antigen. Notably, TCR6 and its derivative TCRT6 is a newly discovered asTCR for NY-ESO-1_(157–165)_ with high specificity and even a higher killing effect than TCRTe, whose affinity had been deliberately enhanced. Our study has also identified 1G4-related TCRs with higher NY-ESO-1 binding and higher cancer cell-killing effect.

Our previous study demonstrated the tumorigenesis (proliferation, progression, metastasis, and angiogenesis) role TRPV2 plays in EC through the HSP70/27 and PI3K signaling pathways [16]. In the same study, we reported the significant tumorigenesis attenuation of EC in vitro and in vivo using Tranilast, an antagonist to TRPV2. In that study, we showed in detail the role TRPV2 plays in EC tumorigenesis and reported the use of Tranilast to antagonize TRPV2, resulting in the growth inhibition of EC cells and tumors. Numerous studies have also reported a direct association between TRPV2 and worse prognosis in EC patients [36–38], inferring the association of TRPV2 expression to the unimproving survival rate of EC patients. Additionally, Tranilast has been shown not to have a detrimental effect on CD8+ Cytotoxic T lymphocytes and no antiproliferative effect on CD4 T lymphocytes at concentrations below 150 μM dose [23], making it a good choice for TCR-T combinatorial treatment. Our TCR-Ts have exhibited a significant EC-killing effect by targeting NY-ESO-1 antigen. Tranilast also revealed a significant antitumor effect on EC (Figs. 4e–g, and 7). Our previous study reported that Tranilast kills EC by antagonizing TRPV2 expressed by EC cells. These two distinct biomarker targets (NY-ESO-1 and TRPV2) presented an avenue for a bivalent therapeutic targeting of EC emanating from TCR-T and Tranilast combinatorial treatment accentuated in this study. Our result indicated that TCR-T—Tranilast combined treatment of different EC yielded significant EC cell killing of up to 85% specific lysis compared to only TCR-T or only Tranilast treatment (Figs. 5e(iii), 6d(iii) and 7). This bivalent treatment is novel for T cell adoptive therapy and, for that matter, novel for EC treatment, although several combinatorial adoptive therapies have been reported targeting NY-ESO-1 antigen [39].

Summarily, we report a new way of identifying asTCRs, which is different from the traditional T cell cloning, TILs or autologous T cell deep sequencing to test the most frequent T cells found in the tumor cells. Our technique route relegates all those trial and luck processes and dawns a systematic yet trustworthy system like the Tomlinson I and J library for antibodies. Our findings showed a promising treatment choice for treating EC using either TCR-T adoptive therapy, Tranilast antitumor therapy, or a combination of both treatments using TCR-T and Tranilast or bivalent treatment. However, this study is limited by not enhancing the binding affinities of the isolated asTCRs to investigate the effect in terms of effector function and off-target toxicity associated with synthetic TCR maturation affinity. Successfully targeting cells with asTCRs with higher affinities in vivo is a prerequisite for a successful treatment due to the likelihood of a lower-density pHLA on tumor surfaces. Although affinities of asTCRs could be enhanced through directed evolution, great attention should be paid to specificity. Another limitation, which will be investigated in a further study, is to examine the killing effect of the TCR-Ts in vivo*,* and prognosticate and investigate potential safety issues or adverse (side) effects. Despite these limitations, the goal of this research, as hypothesized, was to find a cost-effective, quicker, and effective way to isolate asTCRs with inherent higher affinities targeting NY-ESO-1 antigen biomarker in EC and also enhance the antitumor response efficacy through a combinatorial therapy of TCR-T and Tranilast which is unprecedented. TCR-T therapy has shown to be an incredible therapeutic option for cancer patients. Also, we recommend that studies where more antitumor therapies could be combined with TCR-T therapy to yield superior antitumor outcomes should be conducted. Since tumorigenesis is linked with many genes and pathways, more tumor biomarker targets could be screened for, not excluding neoantigens.

## Conclusion

This study reports on a novel way of building a high-quality library that can produce many antigen-specific T cell receptors for a wide range of antigens for T cell adoptive therapy. Hypothesizing the recurrence and over-expression of NY-ESO-1 in esophageal cancer provided an avenue to test the quality and performance of asTCRs isolated from the library through a rigorous screening process. The effector function of the TCR-T cells derived from their asTCRs confirmed our library’s robustness and indicated a therapeutic option using TCR-T therapy. Additionally, our dual-targeted treatment comprising TCR-T and Tranilast showed a superior cancer-killing effect, paving the way for a better cancer (EC) prognosis via combinatorial therapy. Our study suggests an effective, less costly, and quicker way of isolating asTCRS with inherent higher antigen binding affinities, higher anticancer response efficacy, and it can also thrive and yield superior anticancer response when combined with other treatments.

### Supplementary Information

**Additional file 1: Table S1.** Healthy PBMC donors’ demography and HLA genotype. **Table S2.** Degenerate oligonucleotides for TCR alpha variable (TRAV) and TCR beta variable (TRBV) gene amplification and their reverse primers. **Table S3.** Peptide epitopes used for making pHLA. **Table S4.** NY-ESO-1 adoptive T cell therapy in clinical trials. **Table S5.** EC50 values obtained from Tranilast and EC cell lines dose–response curve. **Figure S1.** TCR gene amplification. **a**, Total RNA isolated from CD8+ cytotoxic T lymphocytes obtained from PBMC healthy donors. **b**, Quality assessment cDNA synthesized from the total RNA in **(a)**. The cDNA was used as a template to amplify actin beta (ACTB), beta-2-microglobulin (B2M), and glyceraldehyde-3-phosphate dehydrogenase (GAPDH) housekeeping genes. **c and d,** αTCR and βTCR variable genes amplification using degenerate oligonucleotides. **Figure S2.** Sequence alignment of isolated asTCRs. **Figure S3.** NY-ESO-1 antigen expression in EC cell lines. **a,** NY-ESO-1 antigen expression amplification using cDNA reverse transcribed from mRNA of EC cell lines. **b,** GAPDH housekeeping gene control for EC cell lines. Lanes 1, 2, 3, 4, and 5 represent plasmid containing NY-ESO-1 antigen, ECA109(NY-ESO-1^−^/HLA-A*02:01^−^), COLO680N(NY-ESO-1^−^/HLA-A*02:01^+^), ECA109(NY-ESO-1^+^/HLA-A*02:01^+^), and OE19(NY-ESO-1^+^/HLA-A*02:01^+^) respectively. **Figure S4.** HLA-A2 typing of EC cell lines. EC cell lines that express HLA-A2 allotype were evaluated using HLA-A2 antibody in a flow cytometry analysis. **Figure S5.** Evaluation of cytotoxic T cells expressing asTCR. **a,** cytotoxic CD8+ T cells were quantified from transduced TCR-T population using flow cytometry. TCR-Ts doubling as CD8+ and simultaneously expressing asTCR were gated at the second (upper-right) quadrant with unstained and untransduced T cells as control. **Figure S6.** Puromycin selection dose–response curve. To generate HLA-A*02:01 expressing cells. Puromycin resistance was evaluated using a dose–response curve where 3 μg/ml puromycin inhibited the growth of over 95% of unexpressing HLA-A*02:01 and unresistant puromycin cells in 72 h.

## Data Availability

Data is provided within the manuscript or supplementary materials. All presented data are also available upon request from the corresponding authors.

## Abbreviations

ACTB: Actin beta
ANOVA: Analysis of variance
asTCR: Antigen-specific TCR
B2M: Beta-2-microglobulin
CCK-8: Cell counting kit-8
CDR: Complementarity determining region
EADC: Esophageal adenocarcinoma
EC: Esophageal cancer
EF-1α: Elongation factor 1 alpha
ELISA: Enzyme-linked immunosorbent assay
ESCC: Esophageal squamous cell carcinoma
FBS: Fetal bovine serum
GAPDH: Glyceraldehyde 3-phosphate dehydrogenase
HLA: Human leucocyte antigen
IFNγ: Interferon-gamma
LDH: Lactate dehydrogenase
MOI: Multiplicity of infection
NGS: Next-generation sequencing
NY-ESO-1: New York esophageal squamous cell carcinoma 1
pHLA: Peptide human leucocyte antigen
RFU: Relative fluorescence unit
TAA: Tumor-associated antigen
TCR: T cell receptor
TCR-T: T cell receptor-engineered T cell
TILs: Tumor infiltrating lymphocytes
TRPV2: Transient receptor potential vanilloid type 2
