# Supplementary material for: NY-ESO-1-specific T cell receptor-engineered T cells and Tranilast, a TRPV2 antagonist bivalent treatment enhances the killing of esophageal cancer: a dual-targeted cancer therapeutic route
Source: Cancer Cell Int. 2024 Feb 9;24:64. doi: 10.1186/s12935-024-03249-w (PMC10858587; doi:10.1186/s12935-024-03249-w)
Supplement: Supplementary file 1 — Additional file 1: Table S1. Healthy PBMC donors’ demography and HLA genotype. Table S2. Degenerate oligonucleotides for TCR alpha variable (TRAV) and TCR beta variable (TRBV) gene amplification and their reverse primers. Table S3. Peptide epitopes used for making pHLA. Table S4. NY-ESO-1 adoptive T cell therapy in clinical trials. Table S5. EC50 values obtained from Tranilast and EC cell lines dose–response curve. Figure S1. TCR gene amplification. a, Total RNA isolated from CD8+ cytotoxic T lymphocytes obtained from PBMC healthy donors. b, Quality assessment cDNA synthesized from the total RNA in (a). The cDNA was used as a template to amplify actin beta (ACTB), beta-2-microglobulin (B2M), and glyceraldehyde-3-phosphate dehydrogenase (GAPDH) housekeeping genes. c and d, αTCR and βTCR variable genes amplification using degenerate oligonucleotides. Figure S2. Sequence alignment of isolated asTCRs. Figure S3. NY-ESO-1 antigen expression in EC cell lines. a, NY-ESO-1 antigen expression amplification using cDNA reverse transcribed from mRNA of EC cell lines. b, GAPDH housekeeping gene control for EC cell lines. Lanes 1, 2, 3, 4, and 5 represent plasmid containing NY-ESO-1 antigen, ECA109(NY-ESO-1−/HLA-A*02:01−), COLO680N(NY-ESO-1−/HLA-A*02:01+), ECA109(NY-ESO-1+/HLA-A*02:01+), and OE19(NY-ESO-1+/HLA-A*02:01+) respectively. Figure S4. HLA-A2 typing of EC cell lines. EC cell lines that express HLA-A2 allotype were evaluated using HLA-A2 antibody in a flow cytometry analysis. Figure S5. Evaluation of cytotoxic T cells expressing asTCR. a, cytotoxic CD8+ T cells were quantified from transduced TCR-T population using flow cytometry. TCR-Ts doubling as CD8+ and simultaneously expressing asTCR were gated at the second (upper-right) quadrant with unstained and untransduced T cells as control. Figure S6. Puromycin selection dose–response curve. To generate HLA-A*02:01 expressing cells. Puromycin resistance was evaluated using a dose–response curve where 3 μg/ml puromycin inhibi [file 12935_2024_3249_MOESM1_ESM.docx]

**T cell receptor-engineered T cells and Tranilast, a TRPV2 antagonist bivalent treatment enhances the killing of esophageal cancer: a dual-targeted cancer therapeutic route**

Obed Boadi Amissah^1,2,5*^, Wenfang Chen^1,2^, Jean de Dieu Habimana^1^, Yirong Sun^1^, Lihui Lin^1^, Yujie Liu^1^, Ling Wang^1,3^, Zhaoming Liu^1,2^, Omar Mukama^1^, Rajesh Basnet^1,2^, Hohua Liu^6^, Junyi Li^1,2^, Xuanyan Ding^1,2^, Lingshuang Lv^1,2^, Min Chen^1,3^, Yalin Liang^4^, Rongqi Huang^1,5*^, and Zhiyuan Li^1,2,3,4,5,7*^

^1^CAS Key Laboratory of Regenerative Biology, Guangdong Provincial Key Laboratory of Stem Cell and Regenerative Medicine, Guangzhou Institutes of Biomedicine and Health, Chinese Academy of Sciences, Guangzhou 510530, China.

^2^University of Chinese Academy of Sciences, 19 Yuquan Road, Shijingshan District, Beijing 100049, China.

^3^School of Life Sciences, University of Science and Technology of China, Hefei 230026, China.

^4^GZMU-GIBH Joint School of Life Sciences, Guangzhou Medical University, Guangzhou 511436, China.

^5^GIBH-HKU Guangdong-Hong Kong Stem Cell and Regenerative Medicine Research Centre, GIBH-CUHK Joint Research Laboratory on Stem Cell and Regenerative Medicine, Guangzhou Institutes of Biomedicine and Health, Chinese Academy of Sciences, Guangzhou 510530, China.

^6^Guangdong Provincial Key Laboratory of Protein Function and Regulation in Agricultural Organisms, College of Life Sciences, South China Agricultural University, Guangzhou 510642, China.

^7^Department of Anatomy and Neurobiology, Xiangya School of Medicine, Central South University, Changsha 410013, China.

*Correspondence: Obed Boadi Amissah [obed@gibh.ac.cn](mailto:obed@gibh.ac.cn)

Ronqi Huang [huang_ronqi@gibh.ac.cn](mailto:huang_ronqi@gibh.ac.cn)

Zhiyuan Li [li_zhiyuan@gibh.ac.cn](mailto:li_zhiyuan@gibh.ac.cn)

**Additional Data**

**Additional Tables**

**Table S1.**

**Healthy PBMC donors’ demography and HLA genotype**

Exogenous viruses or their antibodies, such as HIV antibodies, Hepatitis B surface antigen and antibody, Hepatitis B e antigen and antibody, Hepatitis B core antibody, Syphilis (TRUST, TPPA), Hepatitis C antibody, Cytomegalovirus, Epstein-Barr virus, Human T lymphoblastic leukemia virus antibody, and Human parvovirus B19 were tested to ascertain the healthiness of the donors.

| **ID** | **Age** | **Blood type** | **Sex** | **Viral antigen/antibody** | **HLA-A genotype** |
| --- | --- | --- | --- | --- | --- |
| PBCD8-1 | 25 | B+ | Male | Negative | HLA-A*2:01 / A*2:01 |
| PBCD8-2 | 19 | B+ | Male | Negative | HLA-A*2:01 / A*2:01 |
| PBCD8-3 | 42 | A+ | Male | Negative | HLA-A*2:01 / A*2:01 |

**Table S2.**

**Degenerate oligonucleotides for TCR alpha variable (TRAV) and TCR beta variable (TRBV) gene amplification and their reverse primers**

| **TRAV primer** | **Degenerate gene family** | **Oligonucleotide 5’ to 3’** |
| --- | --- | --- |
| TRAV1 | TRAV1-1*01, TRAV1-1*02,  TRAV1-2*01, TRAV1-2*02 | CATGCCATGGGGACAAARCMTTGASCAG |
| TRAV2 | TRAV2*01, TRAV2*02 | CATGCCATGGAAGGACCAAGTGTTTCAG |
| TRAV3 | TRAV3*01, TRAV3*02 | CATGCCATGGGCTCAGTCAGTGGCTCAG |
| TRAV4 | TRAV4*01, TRAV26-1*02,  TRAV26-1*01, TRAV26-1*03,  TRAV26-2*01, TRAV26-2*02 | CATGCCATGGSWTGCTAAGACCACMCAG |
| TRAV5 | TRAV5*01, TRAV13-2*01  TRAV13-2*02, TRAV13-1*03,  TRAV13- 1*02, TRAV13-1*01 | CATGCCATGGGGAGAGRRTGTGGRGCWG |
| TRAV6 | TRAV6*02, TRAV6*01, | CATGCCATGGAGCCAAAAGATAGAACAG |
| TRAV7 | TRAV7*01, TRAV20*03,  TRAV20*04, TRAV20*02,  TRAV20*01 | CATGCCATGGGAARACCAGGTGRMGCAS |
| TRAV8 | TRAV8-5*01, TRAV8-3*03,  TRAV8-3*01, TRAV8-3*02,  TRAV8-7*01, TRAV8-1*01,  TRAV8-1*02, TRAV8-1*02,  TRAV8-6*01, TRAV8-6*02,  TRAV8-2*01, TRAV8-2*02,  TRAV8-4*03, TRAV8-4*05,  TRAV8-4*04, TRAV8-4*01,  TRAV8-4*02 | CATGCCATGGRCCCAGTCDGTGASCCAG |
| TRAV9 | TRAV9-1*01, TRAV9-2*04,  TRAV9-2*01, TRAV9-2*03,  TRAV9-2*02 | CATGCCATGGGGARATTCAGTGRYCCAG |
| TRAV10 | TRAV10*01, TRAV41*01 | CATGCCATGGAAAAAYSAAGTGGAGCAG |
| TRAV11 | TRAV11*01, TRAV15*01 | CATGCCATGGCTMCATAYWCTGGAGYAG |
| TRAV12 | TRAV12-1*01, TRAV12-1*02,  TRAV12-2*01, TRAV12-2*02,  TRAV12-3*01, TRAV12-3*02 | CATGCCATGGCRGAAGGAGGTGGAGCAG |
| TRAV14 | TRAV33*01, TRAV19*01,  TRAV14/DV4*02, TRAV14/DV4*01, TRAV14/DV4*03 | CATGCCATGGGCYCAGAARRTAACYCAA |
| TRAV16 | TRAV38-2/DV8*01, TRAV38-1*04,  TRAV38-1*03, TRAV38-1*01,  TRAV38-1*02, TRAV16*01 | CATGCCATGGGCYCAGASAGTCACTCAG |
| TRAV17 | TRAV17*01 | CATGCCATGGAGTCAACAGGGAGAAGAG |
| TRAV18 | TRAV18*01 | CATGCCATGGGGAGACTCGGTTACCCAG |
| TRAV21 | TRAV21*01, TRAV21*02 | CATGCCATGGAAACAGGAGGTGACRCAG |
| TRAV22 | TRAV22*01 | CATGCCATGGGGAATACAAGTGGAGCAG |
| TRAV23 | TRAV23/DV6*02, TRAV23/DV6*01,  TRAV23/DV6*03 | CATGCCATGGCAGCAGCAGGTGAAACAA |
| TRAV24 | TRAV24*01, TRAV24*02,  TRAV39*01 | CATGCCATGGRWRCTGAAMGTGGAACAA |
| TRAV25 | TRAV25*01 | CATGCCATGGGGACAACAGGTAATGCAA |
| TRAV27 | TRAV27*02, TRAV27*01,  TRAV27*03 | CATGCCATGGACCCAGCTGCTGGAGCAG |
| TRAV28 | TRAV28*01 | CATGCCATGGAAAGTGGAGCAGAGTCCT |
| TRAV29 | TRAV29/DV5*03, TRAV29/DV5*02,  TRAV29/DV5*01 | CATGCCATGGGACCAGCAAGTTAAGCAA |
| TRAV30 | TRAV30*01, TRAV30*02, TRAV30*03, TRAV30*04 | CATGCCATGGCAACAACCAGTGCAGAGT |
| TRAV31 | TRAV31*01 | CATGCCATGGCAGAGGGTCATTCAATCC |
| TRAV32 | TRAV32*01 | CATGCCATGGAAGGATGTGATACAGAGT |
| TRAV34 | TRAV34*01 | CATGCCATGGAGCCAAGAACTGGAGCAG |
| TRAV35 | TRAV35*01, TRAV35*02 | CATGCCATGGGGTCAACAGCTGAATCAG |
| TRAV36 | TRAV36/DV7*02, TRAV36/DV7*01,  TRAV36/DV7*03, TRAV36/DV7*04 | CATGCCATGGGAAGACAAGGTGGTACAA |
| TRAV37 | TRAV37*01 | CATGCCATGGCAACTGCCAGTGGAACAG |
| TRAV40 | TRAV40*01 | CATGCCATGGAGCAATTCAGTCAAGCAG |
| αTCR reverse primer |  | TTTTCCTTTTCTCTCAGCTGGTACACGGCA |
|  | | |
| **TRBV primer** | **Degenerate gene family** | **Oligonucleotide 5’ to 3’** |
| TRBV1 | TRBV1*01, TRBV4-1*01 | CATGCCATGGGAYACTGRARTTACCCAG |
| TRBV2 | TRBV17*01, TRBV2*02,  TRBV2*01, TRBV2*03 | CATGCCATGGGARCCTGRAGTCASCCAG |
| TRBV3 | TRBV3-2*03, TRBV3-2*02,  TRBV3-2*01, TRBV3-1*01,  TRBV3-1*02 | CATGCCATGGGACACAGCCGTTTCCCAG |
| TRBV4 | TRBV4-3*01, TRBV4-3*03,  TRBV4-3*02, TRBV4-2*01,  TRBV4-2*02, TRBV14*01,  TRBV14*02 | CATGCCATGGGAARCKGGAGTTACKCAG |
| TRBV5 | TRBV5-1*02, TRBV5-1*01 | CATGCCATGGARGGCTGGRGTCACTCAA |
| TRBV6 | TRBV6-2*01, TRBV10-2*01,  TRBV6-3*01, TRBV6-2*02,  TRBV6-1*01, TRBV6-7*01,  TRBV6-8*01, TRBV6-9*01,  TRBV6-5*01, TRBV6-6*04,  TRBV6-6*05, TRBV6-6*02,  TRBV6-6*01, TRBV6-6*03 | CATGCCATGGRATGCTGGWRTCACYCAG |
| TRBV7 | TRBV7-1*01, TRBV7-2*02,  TRBV7-2*03, TRBV7-2*01,  TRBV7-2*04, TRBV7-3*01,  TRBV7-3*04, TRBV7-3*03,  TRBV7-5*01, TRBV7-5*02,  TRBV7-4*01, TRBV7-4*02,  TRBV7-8*03, TRBV7-8*01,  TRBV7-8*02, TRBV7-6*01,  TRBV7-6*02, TRBV7-7*01,  TRBV7-7*02, TRBV7-9*05,  TRBV7-9*06, TRBV7-9*03,  TRBV7-9*01, TRBV7-9*02 | CATGCCATGGGRWRCTGGAGTYTCCCAG |
| TRBV8 | TRBV8-1*01, TRBV8-2*01 | CATGCCATGGGAKGCWGGGATCASCCAG |
| TRBV9 | TRBV5-2*01, TRBV5-8*01,  TRBV5-3*01, TRBV5-3*02,  TRBV5-4*01, TRBV5-4*02,  TRBV5-6*01, TRBV5-7*01,  TRBV5-5*03, TRBV5-5*01,  TRBV5-5*02, TRBV9*01,  TRBV9*03, TRBV9*02 | CATGCCATGGGABDCTGGARTCACMCAA |
| TRBV10 | TRBV10-1*01, TRBV10-1*02,  TRBV10-3*01, TRBV10-3*03,  TRBV10- 3*04, TRBV10-3*02,  TRBV6-4*01, TRBV10-2*01,  TRBV6-4*02 | CATGCCATGGRHTGCTGRRATCACCCAG |
| TRBV11 | TRBV11-3*01, TRBV11-3*02,  TRBV11-1*01, TRBV11-2*02,  TRBV11-2*01, TRBV11-2*03 | CATGCCATGGGAAGCTGRAGTKGYYCAG |
| TRBV12 | TRBV12-2*01, TRBV12-1*01,  TRBV12-5*01, TRBV12-3*01,  TRBV12-4*01, TRBV12-4*02,  TRBV11-3*03 | CATGCCATGGGATGCTRGHRTTAYCCAG |
| TRBV13 | TRBV13*01, TRBV13*02 | CATGCCATGGGCTGCTGGAGTCATCCAG |
| TRBV15 | TRBV15*03, TRBV15*01,  TRBV15*02, TRBV21-1*01,  TRBV21/OR9-2*01 | CATGCCATGGGAYRCCAWGGTCAYCCAG |
| TRBV16 | TRBV16*03, TRBV16*01,  TRBV16*02 | CATGCCATGGGGTGAAGAAGTCGCCCAG |
| TRBV17 | TRBV7-9*04 | CATGCCATGGATATCTGGAGTCTCCCAC |
| TRBV18 | TRBV18*01 | CATGCCATGGAATGCCGGCGTCATGCAG |
| TRBV19 | TRBV19*02, TRBV19*03,  TRBV19*01 | CATGCCATGGGATGGTGGAATCACTCAG |
| TRBV20 | TRBV29/OR9-2*01,  TRBV29/OR9-2*02, TRBV29-1*01,  TRBV29-1*02, TRBV20-1*07,  TRBV20-1*05, TRBV20-1*06,  TRBV20-1*04, TRBV20-1*03,  TRBV20-1*01, TRBV20-1*02,  TRBV20/OR9-2*02,  TRBV20/OR9-2*01 | CATGCCATGGRGTGCTGTCRTCTCTCAA |
| TRBV22 | TRBV22-1*01 | CATGCCATGGGATGCTGACATCTATCAG |
| TRBV23 | TRBV23-1*01, TRBV23/OR9-2*01,  TRBV23/ OR9-2*02 | CATGCCATGGCATGCCAAAGTCACACAG |
| TRBV24 | TRBV24-1*01, TRBV24/OR9-2*01,  TRBV24/OR9-2*02,  TRBV24/OR9-2*03 | CATGCCATGGGATGCTGATGTTAYCCAG |
| TRBV25 | TRBV25-1*01, TRBV25/OR9-2*01,  TRBV25/OR9-2*02 | CATGCCATGGGAAGCTGAMATCTACCAG |
| TRBV26 | TRBV26*01, TRBV26/OR9-2*01 | CATGCCATGGGATGCTGTAGTTACACAA |
| TRBV27 | TRBV27*01 | CATGCCATGGGAAGCCCAAGTGACCCAG |
| TRBV28 | TRBV28*01 | CATGCCATGGRATGTSAAAGTAACMCAG |
| TRBV30 | TRBV30*05, TRBV30*01,  TRBV30*02, TRBV30*04 | CATGCCATGGTCTCAGACTATTCATCAA |
| βTCR reverse primer |  | TTTTCCTTTTGCGGCCGCTTCTGATGGCTCAAACACAG |

**Table S3.**

**Peptide epitopes used for making pHLA**

| **No.** | **Peptide** | **Biomarker** | **Epitope** | **NY-ESO-1** | **HLA-A*02:01** | **Relevance** |
| --- | --- | --- | --- | --- | --- | --- |
| 1. | SLLMWITQC | NY-ESO-1 | 157 - 165 | + | + | Relevant |
| 2. | SISSCLQQL | NY-ESO-1 | 148 - 156 | + | + | Irrelevant |
| 3. | FMNKFIYEI | Alpha-fetoprotein (AFP) | 158 - 166 | - | + | Irrelevant |
| 4. | VVGAVGVGK | KRAS G12V | 7 - 16 | - | - | Irrelevant |

**Table S4.**

**NY-ESO-1 adoptive T cell therapy in clinical trials**

| **No.** | **NCT ID** | **Intervention** | **Malignancy** | **Phase** | **Sponsor** |
| --- | --- | --- | --- | --- | --- |
| 1. | [NCT02366546](https://clinicaltrials.gov/ct2/show/NCT02366546) | NY-ESO-1 TCR-T combined with TBI-1301, Cyclophosphamide, or Fludarabine | Unresectable solid tumor | I | Mie University |
| 2. | [NCT02366546](https://clinicaltrials.gov/ct2/show/NCT02366546) | Mouse NY-ESO-1 TCR-T (TBI-1301) | Synovial sarcoma | I | University Health Network, Toronto |
| 3. | [NCT03250325](https://clinicaltrials.gov/ct2/show/NCT03250325) | Mouse NY-ESO-1 TCR-T (TBI-1301) | Synovial sarcoma | II | Takara Bio Inc. |
| 4. | [NCT03047811](https://clinicaltrials.gov/ct2/show/NCT03047811) | NY-ESO-1 c259 autologous T cells | Advanced solid tumors | N/A | Fudan University |
| 5. | [NCT02457650](https://clinicaltrials.gov/ct2/show/NCT02457650) | NY-ESO-1 c259 autologous T cells | Relapsed or refractory malignancies | I | Shenzhen Second People’s Hospital |
| 6. | [NCT01795976](https://clinicaltrials.gov/ct2/show/NCT01795976) | NY-ESO-1 c259 autologous T cells | Advanced esophagogastric cancer | II | Fiona Thistlethwaite, The Christie NHS Foundation Trust |
| 7. | [NCT03093350](https://clinicaltrials.gov/ct2/show/NCT03093350) | NY-ESO-1 c259 autologous T cells | Breast cancer | II | Mothaffar Rimawi, Baylor College of Medicine |
| 8. | [NCT03192462](https://clinicaltrials.gov/ct2/show/NCT03192462) | NY-ESO-1/ multi-TAA specific T cells | Pancreatic cancer | I/II | Benjamin Leon Musher, Baylor College of Medicine |
| 9. | [NCT02291848](https://clinicaltrials.gov/ct2/show/NCT02291848) | NY-ESO-1 c259 autologous T cells and stem cell transplantation | Multiple myeloma | I | Premal Lulla, Baylor College of Medicine |
| 10. | [NCT02494167](https://clinicaltrials.gov/ct2/show/NCT02494167) | NY-ESO-1/ multi-TAA-specific T cells | Acute myeloid leukemia (AML) or myelodysplastic syndrome (MDS) | I | Premal Lulla, Baylor College of Medicine |

**Table S5.**

**EC50 values obtained from Tranilast and EC cell lines dose-response curve**

| **No.** | **Cell line** | **24 h** | **48 h** | **72 h** | **96 h** | **120 h** |
| --- | --- | --- | --- | --- | --- | --- |
| 1. | ECA109 (NY-ESO-1^-^ HLA-A*02:01^-^) | 225.7 | 172.5 | 117.3 | 86.42 | 65.34 |
| 2. | ECA109 (NY-ESO-1^+^ HLA-A*02:01^+^) | 269.8 | 178.6 | 119.5 | 82.08 | 54.84 |
| 3. | OE19 (NY-ESO-1^+^ HLA-A*02:01^+^) | 224.8 | 168.0 | 113.8 | 83.32 | 51.71 |

**Additional Figures**


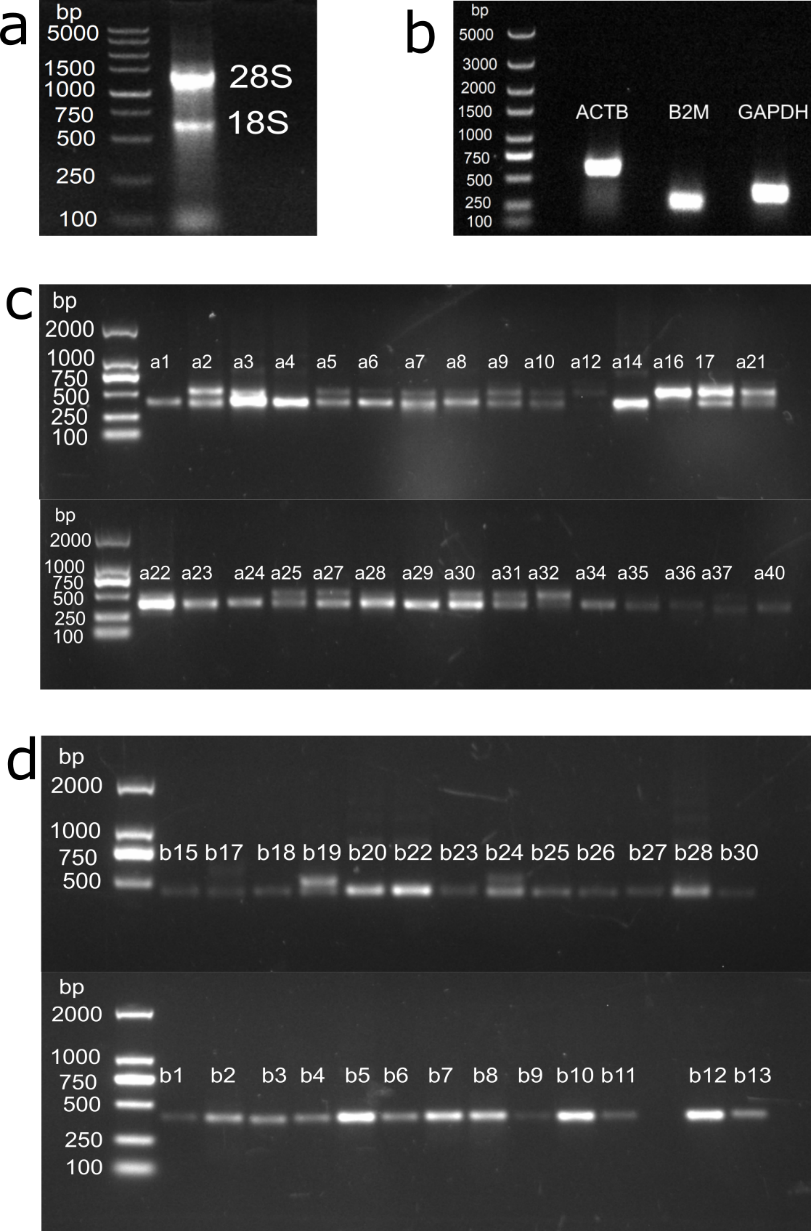


**Figure S1**.

TCR gene amplification. **a**, Total RNA isolated from CD8+ cytotoxic T lymphocytes obtained from PBMC healthy donors. **b**, Quality assessment cDNA synthesized from the total RNA in **(a)**. The cDNA was used as a template to amplify β-actin (ACTB), β-2-microglobulin (B2M), and glyceraldehyde-3-phosphate dehydrogenase (GAPDH) housekeeping genes. **c and d,** αTCR and βTCR variable genes amplification using degenerate oligonucleotides.


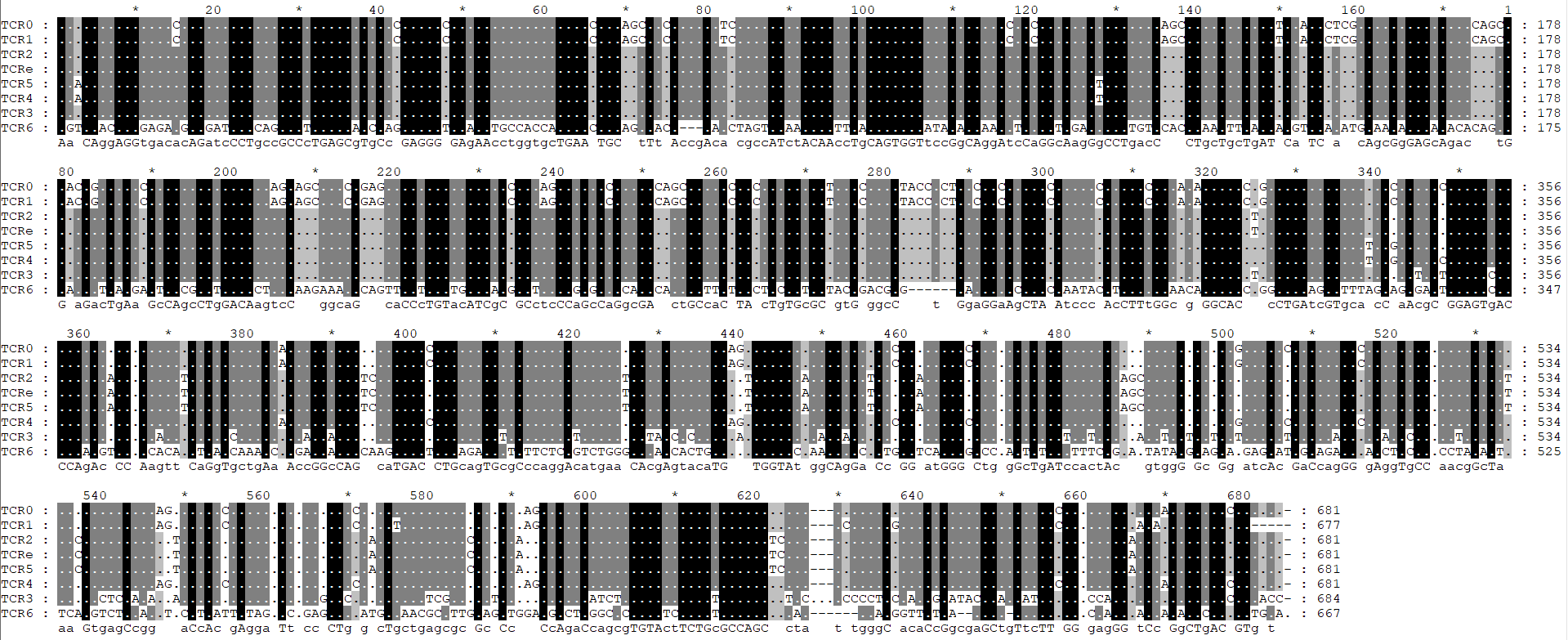


**Figure S2**.

Sequence alignment of isolated asTCRs.


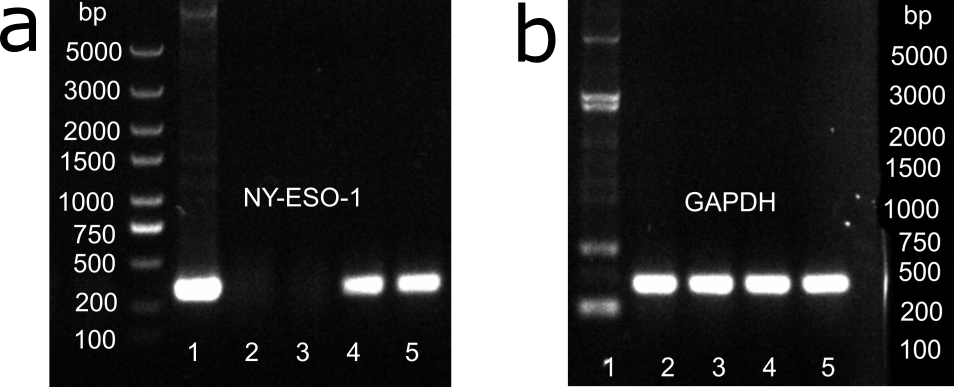


**Figure S3**.

NY-ESO-1 antigen expression in EC cell lines. **a,** NY-ESO-1 antigen expression amplification using cDNA reverse transcribed from mRNA of EC cell lines. **b,** GAPDH housekeeping gene control for EC cell lines. Lanes 1, 2, 3, 4, and 5 represent plasmid containing NY-ESO-1 antigen, ECA109(NY-ESO-1^-^/HLA-A*02:01^-^), COLO680N(NY-ESO-1^-^/HLA-A*02:01^+^), ECA109(NY-ESO-1^+^/HLA-A*02:01^+^), and OE19(NY-ESO-1^+^/HLA-A*02:01^+^) respectively.


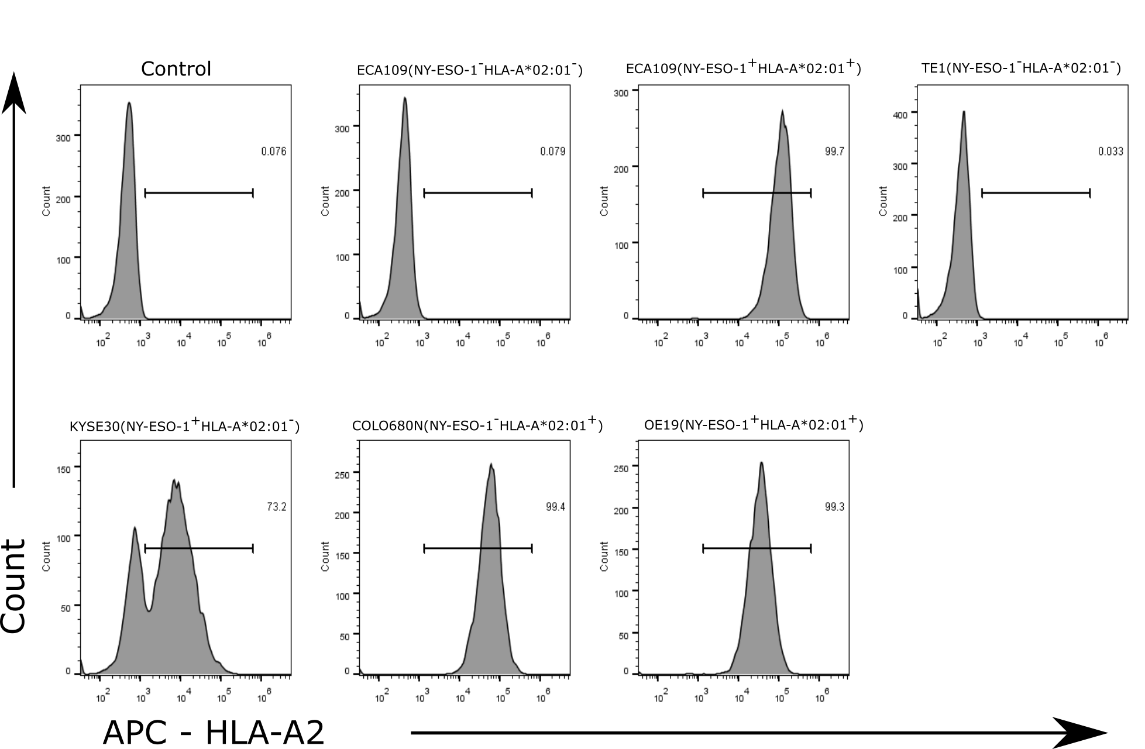


**Figure S4**.

HLA-A2 typing of EC cell lines. EC cell lines that express HLA-A2 allotype were evaluated using HLA-A2 antibody in a flow cytometry analysis.


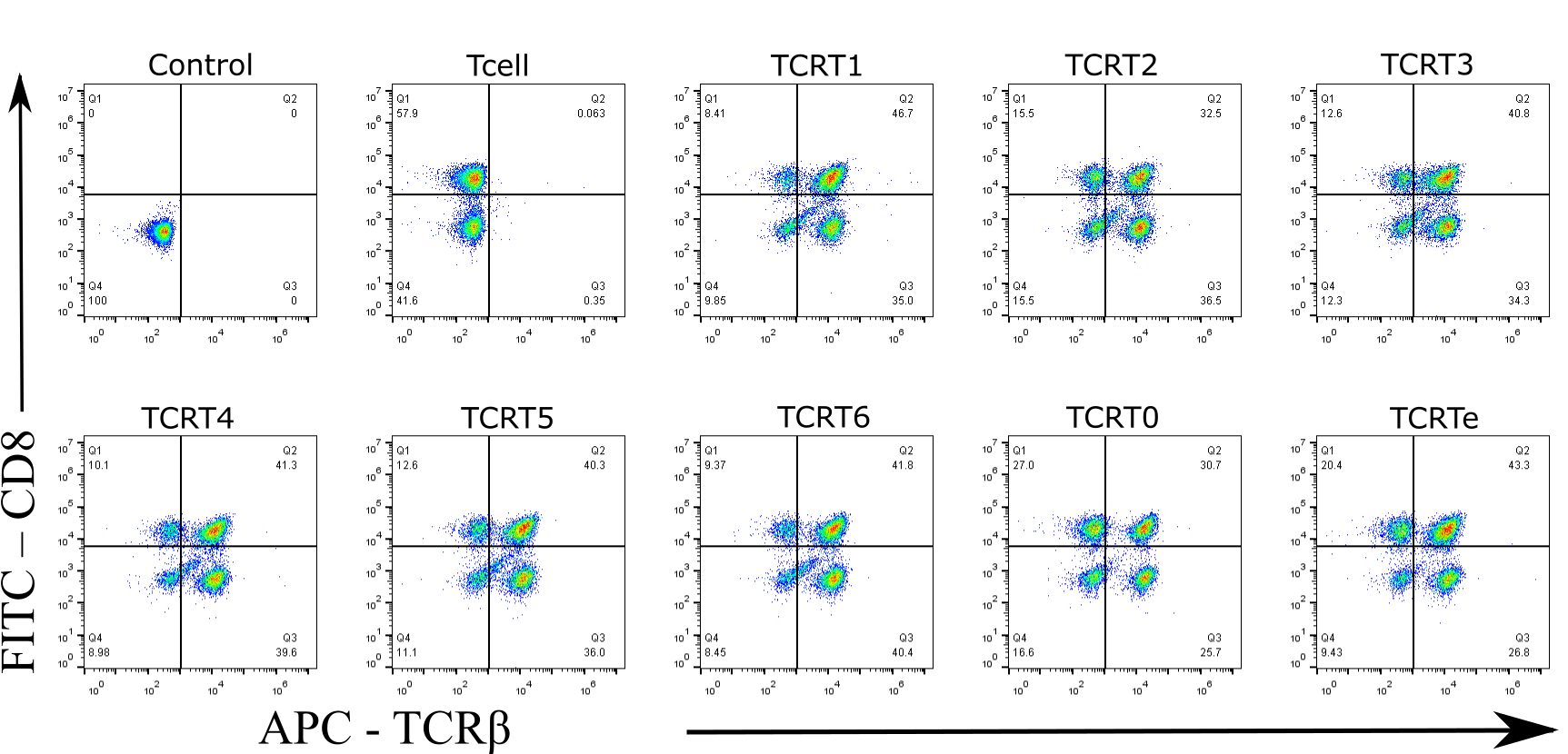


**Figure S5**.

Evaluation of cytotoxic T cells expressing asTCR. **a,** cytotoxic CD8+ T cells were quantified from transduced TCR-T population using flow cytometry. TCR-Ts doubling as CD8+ and simultaneously expressing asTCR were gated at the second (upper-right) quadrant with unstained and untransduced T cells as control.


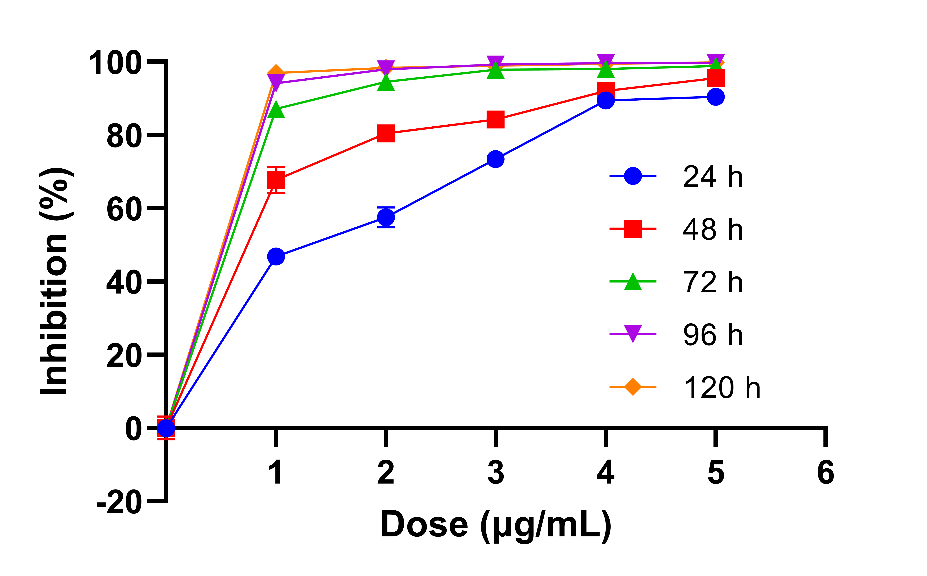


**Figure S6**.

Puromycin selection dose-response curve. To generate HLA-A*02:01 expressing cells. Puromycin resistance was evaluated using a dose-response curve where 3 μg/ml puromycin inhibited the growth of over 95% of unexpressing HLA-A*02:01 and unresistant puromycin cells in 72 h.
